# Projections of temperature-related excess mortality under climate change scenarios

**DOI:** 10.1016/S2542-5196(17)30156-0

**Published:** 2017-12

**Authors:** Antonio Gasparrini, Yuming Guo, Francesco Sera, Ana Maria Vicedo-Cabrera, Veronika Huber, Shilu Tong, Micheline de Sousa Zanotti Stagliorio Coelho, Paulo Hilario Nascimento Saldiva, Eric Lavigne, Patricia Matus Correa, Nicolas Valdes Ortega, Haidong Kan, Samuel Osorio, Jan Kyselý, Aleš Urban, Jouni J K Jaakkola, Niilo R I Ryti, Mathilde Pascal, Patrick G Goodman, Ariana Zeka, Paola Michelozzi, Matteo Scortichini, Masahiro Hashizume, Yasushi Honda, Magali Hurtado-Diaz, Julio Cesar Cruz, Xerxes Seposo, Ho Kim, Aurelio Tobias, Carmen Iñiguez, Bertil Forsberg, Daniel Oudin Åström, Martina S Ragettli, Yue Leon Guo, Chang-fu Wu, Antonella Zanobetti, Joel Schwartz, Michelle L Bell, Tran Ngoc Dang, Dung Do Van, Clare Heaviside, Sotiris Vardoulakis, Shakoor Hajat, Andy Haines, Ben Armstrong

**Affiliations:** a Department of Social and Environmental Health Research, London School of Hygiene & Tropical Medicine, London, UK; b Department of Epidemiology and Preventive Medicine, School of Public Health and Preventive Medicine, Monash University, Melbourne, Australia; c Division of Epidemiology and Biostatistics, School of Population Health, University of Queensland, Brisbane, QLD, Australia; d Potsdam Institute for Climate Impact Research, Potsdam, Germany; e School of Public Health and Institute of Environment and Human Health, Anhui Medical University, Hefei, China; f Shanghai Children's Medical Centre, Shanghai Jiao-Tong University, Shanghai, China; g School of Public Health and Social Work, Queensland University of Technology, Brisbane, QLD, Australia; h Institute of Advanced Studies, University of São Paulo, São Paulo, Brazil; i Department of Epidemiology, Public Health and Preventive Medicine, University of Ottawa, Ottawa, ON, Canada; j Department of Public Health, Universidad de los Andes, Santiago, Chile; k Department of Environmental Health, School of Public Health, Fudan University, Shanghai, China; l Department of Environmental Health, University of São Paulo, São Paulo, Brazil; m Institute of Atmospheric Physics, Academy of Sciences of the Czech Republic, Prague, Czech Republic; n Faculty of Environmental Sciences, Czech University of Life Sciences, Prague, Czech Republic; o Center for Environmental and Respiratory Health Research, University of Oulu, Oulu, Finland; p Medical Research Center Oulu, Oulu University Hospital and University of Oulu, Oulu, Finland; q Santé Publique France, French National Public Health Agency, Saint Maurice, France; r School of Physics, Dublin Institute of Technology, Dublin, Ireland; s Institute of Environment, Health and Societies, Brunel University London, London, UK; t Department of Epidemiology, Lazio Regional Health Service, Rome, Italy; u Department of Pediatric Infectious Diseases, Institute of Tropical Medicine, Nagasaki University, Nagasaki, Japan; v Faculty of Health and Sport Sciences, University of Tsukuba, Tsukuba, Japan; w Department of Environmental Health, National Institute of Public Health, Cuernavaca Morelos, Mexico; x Department of Environmental Engineering, Kyoto University, Kyoto, Japan; y Graduate School of Public Health, Seoul National University, Seoul, South Korea; z Institute of Environmental Assessment and Water Research (IDAEA), Spanish Council for Scientific Research (CSIC), Barcelona, Spain; aa Epidemiology and Environmental Health Joint Research Unit, CIBERESP, University of Valencia, Valencia, Spain; ab Department of Public Health and Clinical Medicine, Umeå University, Umeå, Sweden; ac Department of Clinical Science, Malmö, Lund University, Lund, Sweden; ad Swiss Tropical and Public Health Institute, Basel, Switzerland; ae University of Basel, Basel, Switzerland; af Environmental and Occupational Medicine, National Taiwan University (NTU) and NTU Hospital, Taipei, Taiwan; ag Department of Public Health, National Taiwan University, Taipei, Taiwan; ah Department of Environmental Health, Harvard TH Chan School of Public Health, Boston, MA, USA; ai School of Forestry and Environmental Studies, Yale University, New Haven CT, USA; aj Faculty of Public Health, University of Medicine and Pharmacy of Ho Chi Minh City, Ho Chi Minh City, Vietnam; ak Institute of Research and Development, Duy Tan University, Da Nang, Vietnam; al Environmental Change Department, Centre for Radiation, Chemical & Environmental Hazards, Public Health England, Chilton, UK; am Institute of Occupational Medicine, Edinburgh, UK

## Abstract

**Background:**

Climate change can directly affect human health by varying exposure to non-optimal outdoor temperature. However, evidence on this direct impact at a global scale is limited, mainly due to issues in modelling and projecting complex and highly heterogeneous epidemiological relationships across different populations and climates.

**Methods:**

We collected observed daily time series of mean temperature and mortality counts for all causes or non-external causes only, in periods ranging from Jan 1, 1984, to Dec 31, 2015, from various locations across the globe through the Multi-Country Multi-City Collaborative Research Network. We estimated temperature–mortality relationships through a two-stage time series design. We generated current and future daily mean temperature series under four scenarios of climate change, determined by varying trajectories of greenhouse gas emissions, using five general circulation models. We projected excess mortality for cold and heat and their net change in 1990–2099 under each scenario of climate change, assuming no adaptation or population changes.

**Findings:**

Our dataset comprised 451 locations in 23 countries across nine regions of the world, including 85 879 895 deaths. Results indicate, on average, a net increase in temperature-related excess mortality under high-emission scenarios, although with important geographical differences. In temperate areas such as northern Europe, east Asia, and Australia, the less intense warming and large decrease in cold-related excess would induce a null or marginally negative net effect, with the net change in 2090–99 compared with 2010–19 ranging from −1·2% (empirical 95% CI −3·6 to 1·4) in Australia to −0·1% (−2·1 to 1·6) in east Asia under the highest emission scenario, although the decreasing trends would reverse during the course of the century. Conversely, warmer regions, such as the central and southern parts of America or Europe, and especially southeast Asia, would experience a sharp surge in heat-related impacts and extremely large net increases, with the net change at the end of the century ranging from 3·0% (−3·0 to 9·3) in Central America to 12·7% (−4·7 to 28·1) in southeast Asia under the highest emission scenario. Most of the health effects directly due to temperature increase could be avoided under scenarios involving mitigation strategies to limit emissions and further warming of the planet.

**Interpretation:**

This study shows the negative health impacts of climate change that, under high-emission scenarios, would disproportionately affect warmer and poorer regions of the world. Comparison with lower emission scenarios emphasises the importance of mitigation policies for limiting global warming and reducing the associated health risks.

**Funding:**

UK Medical Research Council.

## Introduction

Climate change is now widely recognised as the biggest global threat of the 21st century.^1^ The Fifth Assessment Report^2^ of the Intergovernmental Panel on Climate Change (IPCC), the leading international body for the assessment of climate change, has established that anthropogenic emissions of greenhouse gases represent the dominant cause for the warming of the planet. Scenarios of climate conditions depend therefore on current and future trajectories of greenhouse gas emissions, mainly determined by socioeconomic development and climate policies.^3^ High-end scenarios, in which no mitigation strategies are in place, predict an average increase in surface temperature between 2·6°C and 4·8°C by the end of this century (2081–2100) relative to 1986–2005.^2^

Impacts on human health can occur through multiple pathways.^4^, ^5^ In addition to indirect effects mediated, for instance, by the spread of disease vectors, increase in food insecurity, and migration and conflicts, direct effects are expected from the increase in extreme weather events such as floods, droughts, and heatwaves.^1^, ^4^ Several studies have focused on the health consequences directly associated with variation in outdoor temperature, predicting an increase in heat-related mortality and morbidity, and—when considered—a concomitant decrease in cold-related mortality.^6^, ^7^, ^8^, ^9^, ^10^, ^11^, ^12^, ^13^ However, evidence on this direct impact at the global scale is limited. This is mainly due to the complexity of modelling the epidemiological relationships, characterised by differential patterns of non-linear and lagged effects associated with heat and cold, and to limitations of previous location-specific or country-specific assessments to capture the heterogeneity of the risk across different populations and climates.^14^, ^15^ Questions also remain about the extent to which expected decreases in cold-related mortality can offset the increase in deaths caused by heat. These issues make it difficult to draw a comprehensive picture of the direct impact of climate change across regions of the world and under different scenarios. This evidence is nonetheless crucial to develop coordinated and evidence-based climate and public health policies.

**Research in context**

**Evidence before this study**

Several studies have evaluated the potential direct health impacts of climate change through variation in temperature-related excess mortality. Most of these investigations have only analysed heat-related impacts, and report an increase in excess mortality proportional to the extent of global warming under different climate change scenarios. Some studies have examined and compared variations in both heat-related and cold-related deaths. As expected, they consistently report an increase in the former and a reduction in the latter. However, results on the net impact on excess mortality are dependent on location and scenarios, and a quantitative comparison is made difficult by the variety of analytical designs that involve alternative effect summaries, statistical modelling, and assumptions.

**Added value of this study**

Our assessment provides a consistent comparison across hundreds of locations in various regions of the world, characterised by different climates, socioeconomic and demographic conditions, and levels of development of infrastructures and public health services. The analysis makes use of advanced analytical methods to flexibly account for changes in both heat-related and cold-related excess mortality, and to take into account local climates and temperature–mortality relationships.

**Implications of all the available evidence**

This study indicates that, in high-emission scenarios, most regions are projected to experience a steep rise in heat-related mortality that will not be equalled by a reduction in cold-related deaths, resulting in a substantial positive net increase in mortality. However, the potential impact varies across areas, and populations living in warmer and potentially poorer regions are expected to sustain an increased burden. Furthermore, the increase in temperature-related excess mortality would be substantially reduced in scenarios involving mitigation strategies to limit greenhouse gas emissions and further warming of the planet, and stricter mitigation approaches are associated with larger benefits. This evidence is crucial for the development of coordinated and evidence-based climate and public health policies, and for informing the ongoing international discussion on the health impacts of climate change.

In this contribution, we present projections of the impact of climate change on temperature-attributable mortality in hundreds of locations around the globe, using recently developed study designs and statistical methods.

## Methods

### Data sources and scenario models

A detailed description of the data, analytical framework, and statistical methods, partly described in previous work,^16^ is provided in the appendix.

We estimated location-specific associations using observed data on outdoor temperature and mortality. For this purpose, we obtained information from a dataset created through the Multi-Country Multi-City (MCC) Collaborative Research Network. The dataset is composed of observed daily time series of mean temperature and mortality counts for all causes or non-external causes only (International Classification of Diseases [ICD] codes 0–799 in ICD-9 and codes A00-R99 in ICD-10) in largely overlapping periods ranging from Jan 1, 1984, to Dec 31, 2015, in addition to location-specific meta-variables (appendix).

We computed future effects under alternative climate change scenarios using modelled climate and mortality projections. First, we obtained daily mean temperature series under scenarios of climate change consistent with the four representative concentration pathways (RCPs) defined in the 2014 IPCC report.^2^ These four scenarios (RCP2.6, RCP4.5, RCP6.0, and RCP8.5) correspond to increasing greenhouse gas concentration trajectories, and describe a range of changes in climate and related global warming, from mild (RCP2.6) to extreme (RCP8.5). We generated the temperature series under each RCP by general circulation models (GCMs), which offer a representation of past, current, and future climate dependent on greenhouse gas emissions. Specifically, projections for five GCMs, representative of the range of available climate models, were developed and made available by the Inter-Sectoral Impact Model Intercomparison Project (ISI-MIP).^17^ The ISI-MIP database provides daily mean temperature for historical (1960–2005) and projected (2006–99) periods, bias-corrected and downscaled at a 0·5° × 0·5° spatial resolution, as single runs of each GCM under each RCP. We extracted the modelled daily temperature series for each of the studied locations in the period 1990–2099 by linking the coordinates with the corresponding cell of the grid, and recalibrated the modelled series using the observed series.^18^ We computed projected daily series of all-cause mortality as the average observed counts for each day of the year, repeated along the same projection period (1990–2099).

### Estimation of the exposure–response relationships

We obtained location-specific estimates of temperature–mortality associations from a two-stage time series analysis, as previously described.^16^ Briefly, in the first stage, we performed a quasi-Poisson regression separately in each location, controlling for season, long-term trends, and day of the week. We modelled the non-linear and delayed exposure–lag–response relationship between temperature and mortality with a distributed lag non-linear model, applying a bidimensional cross-basis spline function with 21 days of lag.^19^ We replaced the quadratic B-spline for the exposure–response relationship used in the previous analysis with a natural cubic spline, which allows a log-linear extrapolation beyond the observed temperature range.

In the second stage, we pooled the reduced estimates of the overall cumulative exposure–response curves using a multivariate meta-regression.^20^ We included a set of meta-predictors to capture part of the heterogeneity across locations: specifically indicators for region, indicators for climate classification,^21^ country-level gross domestic product per capita, and location-specific average and range of temperature. We then derived the best linear unbiased prediction of the overall cumulative exposure–response association in each location, expressed as relative risk.

### Projection of the impact on mortality

We computed the excess mortality attributable to temperature by projecting the impact using the modelled daily series of temperature and mortality under the assumption of no adaptation or population changes, extending a method previously illustrated.^16^ Briefly, for each location, we used the overall cumulative relative risk corresponding to each day's temperature to compute the attributable deaths and fraction in the next 21 days, using the minimum mortality temperature, referred to as the optimal temperature, as the reference. The sum of the contributions from all the days of the series is interpreted as the total excess mortality attributed to non-optimal temperature, whereas the components attributable to cold and heat were separated by summing the subsets corresponding to days with temperatures lower or higher than the minimum mortality temperature, respectively (see appendix for an illustrative example).

We first calculated the excess mortality separately for each location and combinations of GCMs and RCPs. We then computed attributable fractions as GCM-ensemble averages by aggregating by region and country, decade, and RCP, using the related total number of deaths as denominator. We used Monte Carlo simulations to obtain empirical CIs (eCIs), quantifying the uncertainty in both the estimation of the exposure–lag–response relationships and climate projections across GCMs (appendix).

We did all analyses with R (version 3.4.0), using the packages dlnm and mvmeta. The code is available on request from the first author (AG).

### Role of the funding source

The funder of the study had no role in study design, data collection, data analysis, data interpretation, or writing of the report. The corresponding author had full access to all the data in the study and had final responsibility for the decision to submit for publication after obtaining approval from all coauthors.

## Results

**Figure 1:**
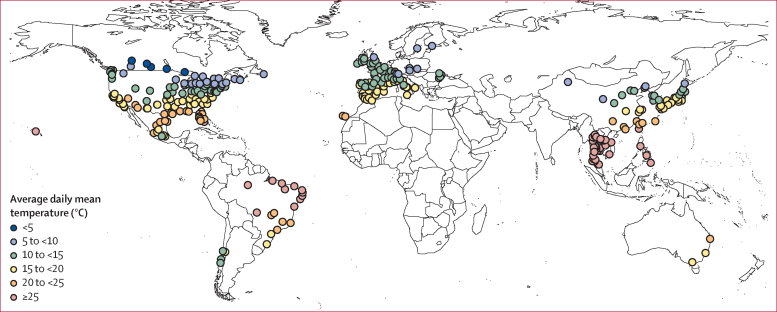
Map of the 451 locations included in the analysis The locations represent metropolitan areas, provinces, or larger areas from 23 countries within nine regions. The colours represent different ranges of average daily mean temperature, computed over the study periods shown in table 1.

We analysed MCC data for 451 locations within 23 countries aggregated in nine regions (separated considering climatic and socioeconomic criteria, and consistent with United Nations geoscheme): North America, Central America, South America, northern Europe, central Europe, southern Europe, east Asia, southeast Asia, and Oceania (referred to from this point on as Australia, which was the only country included in the region; table 1). The dataset included 85 879 895 deaths observed within overlapping periods. The geographical distribution and average mean temperature of the 451 locations shows the wide range of regions of the world included in this assessment and characterised by different climatic conditions, from cold places in North America and northern Europe to tropical areas in South America and southeast Asia (figure 1). However, entire regions of the world, such as Africa and the Middle East, are not represented.

**Table 1:** Descriptive statistics by region and country

	**Number of locations**	**Study period**	**Total deaths**	**Temperature, °C**
**North America**				
Canada	26	1986–2011	2 989 901	6·8 (2·6–10·7)
USA	135	1985–2009	22 953 896	14·9 (7·9–25·5)
**Central America**				
Mexico	10	1998–2014	2 980 086	18·8 (13·9–23·3)
**South America**				
Brazil	18	1997–2011	3 401 136	24·6 (17·7–27·4)
Chile	4	2004–14	325 462	13·7 (11·5–15·4)
**Northern Europe**				
Finland	1	1994–2011	130 325	6·2 (6·2–6·2)
Ireland	6	1984–2007	1 058 215	9·7 (9·1–10·6)
Sweden	1	1990–2002	190 092	7·5 (7·5–7·5)
UK	10	1990–2012	12 075 623	10·3 (9·5–11·6)
**Central Europe**				
Czech Republic	4	1994–2015	711 910	9·1 (8·3–9·9)
France	18	2000–10	1 197 555	12·6 (10·6–16·2)
Moldova	4	2001–10	59 906	10·7 (10·2–11·3)
Switzerland	8	1995–2013	243 638	10·4 (8·6–12·9)
**Southern Europe**				
Italy	11	1987–2010	820 390	15·4 (12·2–18·4)
Spain	52	1990–2014	3 017 110	15·5 (10·9–21·6)
**East Asia**				
China	15	1996–2008	950 130	15·1 (7·4–23·7)
Japan	47	1985–2012	26 893 197	15·3 (9·1–23·1)
South Korea	7	1992–2010	1 726 938	13·7 (12·5–14·9)
**Southeast Asia**				
Philippines	4	2006–10	274 516	28·2 (28·0–28·8)
Taiwan	3	1994–2007	765 893	24·0 (23·2–25·2)
Thailand	62	1999–2008	1 827 853	27·6 (25·1–29·3)
Vietnam	2	2009–13	108 173	27·1 (25·7–28·5)
**Australia**				
Australia	3	1988–2009	1 177 950	18·1 (15·7–20·3)

Temperatures are average location-specific daily mean temperature (range).

Table 2 shows the distribution of average location-specific temperature in the current period (2010–19) and the projected increase at the end of this century (2090–99) under the four climate change scenarios, with a graphical representation of the temperature trends in the appendix. A steep increase is consistently projected under high-end scenarios (RCP6.0 and RCP8.5), whereas in pathways that assume mitigation policies to limit greenhouse gas emissions (RCP2.6 and RCP4.5), the increase slows at different times during the next decades and potentially decreases in some regions under RCP2.6 (appendix). By the end of the century, a reduction in greenhouse gas emissions could prevent a large part of warming in the analysed areas, with the average temperature increase being in the range 0·4–0·8°C under RCP2.6 compared with 3·3–4·9°C under RCP8.5. However, comparison between regions reveals strong geographical differences, with a smaller temperature increase in regions such as Australia and northern Europe compared with southern Europe and South and North America (table 2, appendix).

**Table 2:** Current temperature and projected increase (°C) by RCP and region

	**Current temperature (2010–19)**	**Projected increase (2090–99 *vs* 2010–19)**			
		RCP2.6	RCP4.5	RCP6.0	RCP8.5
North America	14·2 (3·4–26·0)	0·8 (0·5–1·2)	2·2 (1·3–3·0)	2·8 (1·8–3·6)	4·9 (3·2–6·3)
Central America	19·0 (14·1–23·5)	0·6 (0·4–1·0)	1·9 (1·7–2·3)	2·6 (2·3–3·3)	4·5 (4·1–5·4)
South America	22·8 (11·8–27·8)	0·5 (0·3–0·7)	1·5 (1·0–2·0)	1·9 (1·4–2·6)	3·7 (2·8–5·1)
Northern Europe	10·2 (6·9–12·0)	0·5 (0·4–1·1)	1·4 (1·1–2·4)	2·1 (1·6–3·3)	3·4 (2·8–5·4)
Central Europe	11·8 (8·7–16·5)	0·7 (0·4–1·0)	1·8 (1·5–2·0)	2·4 (2·1–2·6)	4·3 (3·5–4·8)
Southern Europe	15·9 (11·3–21·9)	0·7 (0·6–0·8)	1·9 (1·3–2·2)	2·5 (1·8–2·7)	4·5 (3·0–5·1)
East Asia	15·6 (7·6–24·1)	0·7 (0·4–1·1)	1·9 (1·4–2·6)	2·5 (1·7–3·2)	4·3 (3·1–6·0)
Southeast Asia	27·8 (23·6–29·6)	0·6 (0·4–0·8)	1·5 (1·2–1·7)	2·0 (1·7–2·3)	3·8 (3·2–4·3)
Australia	18·5 (16·1–20·7)	0·4 (0·2–0·6)	1·2 (1·1–1·3)	1·8 (1·6–1·9)	3·3 (3·2–3·6)

Data are average mean location-specific temperature (range) as GCM-ensemble. RCP=representative concentration pathway. GCM=general circulation model.

**Figure 2:**
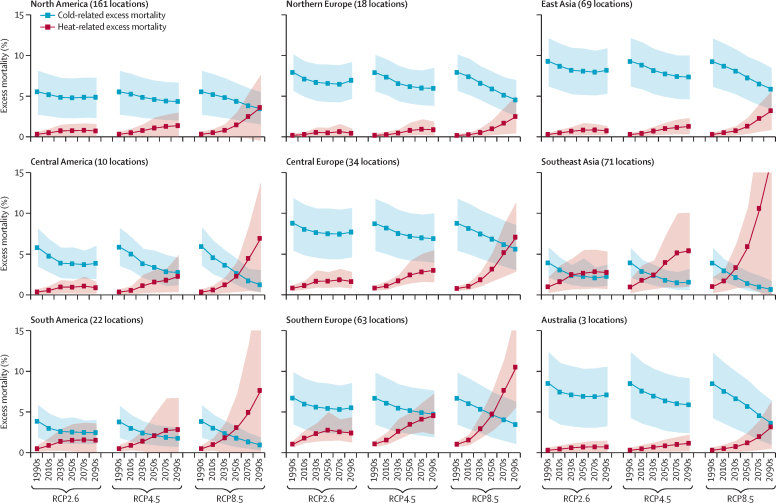
Trends in heat-related and cold-related excess mortality by region The graph shows the excess mortality by decade attributed to heat and cold in nine regions and under three climate change scenarios (RCP2.6, RCP4.5, and RCP8.5). Estimates are reported as GCM-ensemble average decadal fractions. The shaded areas represent 95% empirical CIs. RCP=representative concentration pathway. GCM=general circulation model.

Heat-related and cold-related excess mortality in the nine regions projected under three different RCPs are reported in figure 2 (see appendix for the actual figures by region and country for all the RCPs). As expected, the graphs indicate a common pattern of attenuation in cold-related mortality and rise in the excess associated with heat. Slopes are steeper under RCP8.5, whereas the projected trends slow down during the 21st century under scenarios involving mitigation strategies. The graphs show important differences across regions. In some areas, such as northern Europe, east Asia, and Australia, the currently high cold-related excess mortality is projected to decrease from 7·4–8·7% in 2010–19 to 3·7–5·9% in 2090–99 under scenarios of intense warming (RCP8.5). The heat-related excess mortality is currently low in these regions (0·3–0·5%), and it is projected to increase moderately in the same period and scenario (2·5–3·2%).

By contrast, areas dominated by hotter climates, such as Central and South America, southern Europe, and southeast Asia, show a different pattern and an increased impact of climate change. These regions are currently characterised by relatively higher heat-related impacts, in the order of 0·6–1·7% in 2010–19. The excesses are projected to rise considerably by the end of the century under RCP8.5, reaching 10·5% (95% eCI 5·6 to 17·3) in southern Europe and 16·7% (−1·7 to 33·2) in southeast Asia. Conversely, the cold component becomes less important and would almost disappear in equatorial areas, for instance decreasing to 0·7% (0·1 to 1·7) in southeast Asia at the end of the century. North America and central Europe, regions characterised by diverse climatic conditions or a continental climate with cold winters and relatively hot summers, show results that are intermediate between the two groups.

**Figure 3:**
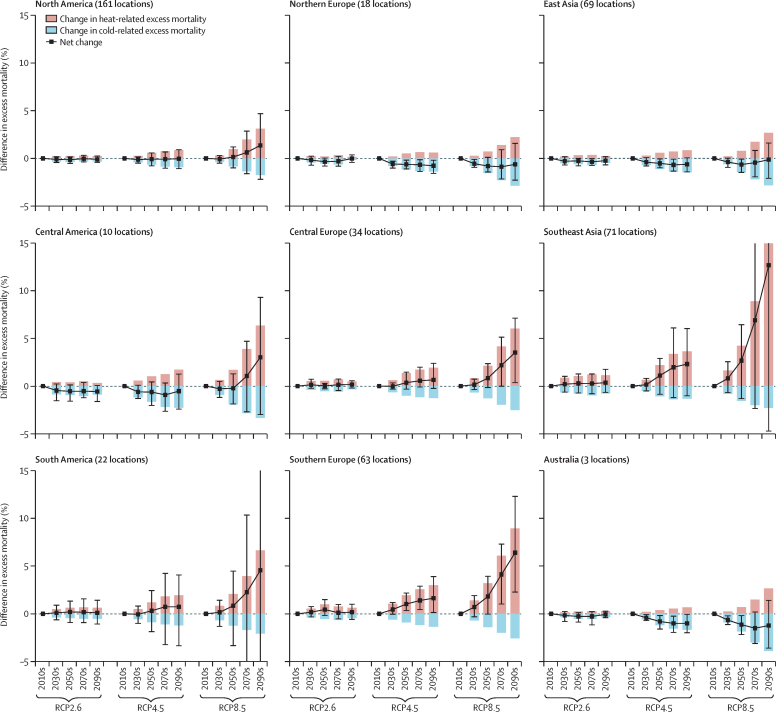
Temporal change in excess mortality by region The graph shows the difference in excess mortality by decade compared with 2010–19 in nine regions and under three climate change scenarios (RCP2.6, RCP4.5, and RCP8.5). Estimates are reported as GCM-ensemble averages. The black vertical segments represent 95% empirical CIs of net difference. RCP=representative concentration pathway. GCM=general circulation model.

With regard to net change in mortality totally attributable to non-optimal temperature (ie, combining heat and cold contributions), the first group of regions (northern Europe, east Asia, and Australia) are projected to initially experience a net reduction, with the net change ranging from −1·2% (95% eCI −3·6 to 1·4) in Australia to −0·1% (−2·1 to 1·6) in east Asia (appendix); however, this pattern would reverse at some point during this century under the more extreme RCP8.5 scenario (figure 3). Conversely, the change in all the other regions, especially those characterised by hotter climates, is driven by the sharp surge in heat-related mortality, with indications of a substantial net increase in excess mortality. The net change becomes pronounced in areas such as South America (4·6% increase, 95% eCI −17·1 to 18·6), southern Europe (6·4% increase, 2·3 to 12·3), Central America (3·0% increase, −3·0 to 9·3), central Europe (3·5% increase, 0·4 to 7·1), and particularly southeast Asia (12·7% increase, −4·7 to 28·1) under RCP8.5 (appendix). Country-specific estimates indicate within-region differences, especially in areas with diverse climates (appendix).

The comparison of the impact across RCPs suggests that the net excess mortality would be reduced under lower greenhouse gas emission scenarios (figure 3). Although an important net increase is still present in hotter areas under RCP4.5, the changes are comparatively very small under the stricter RCP2.6 (figure 3). However, the estimates of the net change are affected by a low precision, due to the uncertainty related to the projected changes in temperature across GCMs and to the extrapolated exposure–response relationships, in particular in areas projected to experience a substantial shift in temperature (appendix).

## Discussion

To our knowledge, this study represents by far the largest epidemiological investigation of potential health effects directly associated with variation in outdoor temperature under climate change scenarios. The assessment includes and compares results from hundreds of locations across various regions of the world, characterised by different climates, socioeconomic and demographic conditions, and levels of development of infrastructures and public health services. The analysis applies advanced analytical methods to flexibly account for changes in both heat-related and cold-related excess mortality, and allows for local climates and temperature–mortality relationships in projecting impacts under different ranges of temperature increase consistent with scenarios of greenhouse gas emissions.

Results of this investigation show that climate change has the potential to produce a substantial increase in temperature-related mortality in most regions. Figures show a steep rise in heat-related excess mortality that, under extreme scenarios of global warming, is not balanced by a decrease in cold-related deaths. However, the predicted impacts show a strong geographical variability. Some temperate areas such as northern Europe, east Asia, and Australia, are characterised by a relatively small projected warming and increase in heat-related mortality. In these regions, the cold component remains higher and the net change would be smaller than in the other regions studied. By contrast, all the other regions are projected to experience a strong surge in heat-related excess mortality, while the cold component becomes progressively less important. The net impact seems to be stronger in warmer areas of America and Europe, and particularly in places with tropical climates such as southeast Asia. Notably, arid or equatorial regions, although under-represented in our dataset, include a large proportion of the current and projected global population, and will contribute greatly to the global impact of climate change.

Changes in temperature-related excess mortality are also highly dependent on the extent of warming expected under alternative emission scenarios. The strongest effects are projected under RCP8.5, a scenario characterised by unabated greenhouse gas emissions and an associated steep increase in temperature. Conversely, the effects of climate change, and particularly the increase in heat-related mortality in warmer regions, are comparatively smaller in scenarios assuming mitigation strategies, and null or marginally negative under the stricter RCP2.6. These findings emphasise the importance of implementation of effective climate policies to contain global warming and prevent the associated negative impacts.

Our results are largely consistent with published investigations in single locations or countries, although previous findings have often been limited to heat-related mortality and are dependent on the choice of location, scenarios, and modelling approaches.^6^, ^7^, ^8^, ^9^, ^10^, ^11^, ^12^, ^13^ In particular, the variety of analytical designs, with alternative effect summaries, statistical modelling, and assumptions, makes it difficult to quantitatively compare results and to draw a comprehensive picture of the global impact of climate change directly attributable to changes in non-optimal temperature exposure. By contrast, our assessment applies an advanced and well tested statistical framework across various regions and climates, accounting for location-specific non-linear and lagged temperature–mortality relationships,^22^ and provides a consistent overview of geographical and temporal differences.

Some assumptions and limitations must be acknowledged. Our projections of current estimates of temperature–mortality associations under future warming scenarios allow isolation of the effects of the changing climate, but ignore contributions from other factors, including demographic changes and adaptation (see appendix).^23^, ^24^, ^25^, ^26^ The reported figures should therefore be interpreted as potential impacts under well defined but hypothetical scenarios, and not as predictions of future excess mortality. We did not choose locations and countries following a sampling procedure that ensured representativeness for each region, and as mentioned above, this study does not provide evidence for large areas of the world owing to insufficient data. Estimates are also affected by considerable uncertainty, particularly those related to the net impact, due to both variability in the climate models and imprecision in the estimated exposure–response curves.^15^ The latter component is often larger, and mainly related to uncertainty in extrapolation of the functions beyond the observed temperature range. In relation to this point, the log-linear extrapolation applied here can be inadequate to pick potential non-linear increases in risk due to particularly intense heat events that might occur in the future, and this would result in an underestimation of heat-related excess deaths.

In summary, this study offers a comprehensive characterisation of climate change impacts due to changes in exposure to non-optimal outdoor temperature, hot as well as cold, across various regions and under alternative scenarios of global warming. Two results must be highlighted. First, the impact varies across areas, and populations living in warmer and, in some cases, poorer regions are expected to experience a heavier burden. Second, increases in temperature-related excess mortality are substantially reduced in scenarios involving mitigation strategies to limit greenhouse emissions and further warming of the planet, and stricter mitigation approaches are associated with larger benefits. The evidence produced in this study can inform the ongoing international discussion and implementation of the recent agreement reached in Paris,^27^, ^28^ and contribute to the development of coordinated and evidence-based climate and public health policies.^1^, ^29^
