# Supplementary material for: Projections of temperature-related excess mortality under climate change scenarios
Source: Lancet Planet Health. 2017 Dec;1(9):e360–7. doi: 10.1016/S2542-5196(17)30156-0 (PMC5729020; doi:10.1016/S2542-5196(17)30156-0)
Supplement: Supplementary appendix [file mmc1.pdf]

THE LANCET
Planetary Health

Supplementary appendix

This appendix formed part of the original submission and has been peer reviewed. We post it as supplied by the authors.

Supplement to: Gasparrini A, Guo Y, Sera F, et al. Projections of temperature-related excess mortality under climate change scenarios. *Lancet Planet Health* 2017; published online Nov 13. http://dx.doi.org/10.1016/S2542-5196(17)30156-0.

Supplementary Appendix
Projections of temperature-related excess mortality under climate change scenarios

Antonio Gasparrini, Yuming Guo, Francesco Sera, Ana Maria Vicedo-Cabrera, Veronika Huber, Shilu Tong, Micheline de Sousa Zanotti Stagliorio Coelho, Paulo Hilario Nascimento Saldiva, Eric Lavigne, Patricia Matus Correa, Nicolas Valdes Ortega, Haidong Kan, Samuel Osorio, Jan Kyselý, Aleš Urban, Jouni J. K. Jaakkola, Niilo R. I. Ryti, Mathilde Pascal, Patrick G. Goodman, Ariana Zeka, Paola Michelozzi, Matteo Scortichini, Masahiro Hashizume, Yasushi Honda, Magali Hurtado-Diaz, Julio Cesar Cruz, Xerxes Seposo, Ho Kim, Aurelio Tobias, Carmen Iñiguez, Bertil Forsberg, Daniel Oudin Åström, Martina S. Ragettli, Yue Leon Guo, Chang-fu Wu, Antonella Zanobetti, Joel Schwartz, Michelle L. Bell, Tran Ngoc Dang, Dung Do Van, Clare Heaviside, Sotiris Vardoulakis, Shakoor Hajat, Andy Haines, Ben Armstrong

Content **Page**

1. Updated information on the MCC dataset 2
2. Detailed information on the analytical framework 5
3. Illustration of the modelling procedure in two cities (with Supplementary Figure S1) 7
4. Supplementary Tables S1-S2 8
5. Supplementary Figures S2-S4 12
6. References 14

1. Updated information on the MCC dataset

The dataset has been collected through the Multi-City Multi-Country (MCC) network, an international collaboration of research teams working on a program aiming to produce epidemiological evidence on associations between weather and health (http://mccstudy.lshtm.ac.uk/). The dataset collects time series data on environmental and health variables, together with metadata, from several locations (either cities, metropolitan areas, provinces, prefectures, or regions) in multiple countries. The current analysis includes data from 451 locations from 23 countries. The datasets from 13 countries (Australia, Brazil, Canada, China, Italy, Japan, South Korea, Spain, Sweden, Taiwan, Thailand, UK, and USA) were described in the online appendix of a previous publication.[1] Here we provide details on datasets from ten additional countries (Chile, Czech Republic, Finland, France, Ireland, Mexico, Moldova, Philippines, Switzerland, and Vietnam) and updates on those from three of the countries previously described (Canada, Spain, and UK).

Canada. We collected data from 25 census metropolitan areas (CMA) and the city of Hamilton between the 1st of January 1986 and 31st of December 2011. A list of 20 CMA is reported in the online appendix of a previous publication.[1] The other five CMA are Niagara, Oakville, Oshawa, Sarnia, and Sault Ste Marie. Daily mortality, obtained from Statistics Canada through access to the Canadian Mortality Database, is represented by counts of deaths for all causes. Mean daily temperature (in ˚C) and relative humidity (in %), computed as the 24-hour average based on hourly measurements, were obtained from Environment Canada. A single weather station was selected for each city using the airport monitoring station located closest to the CMA centre. Measures of ozone (O_3, in ppb), nitrogen dioxide (NO_2, in ppb) and particles ($PM_{2·5}$, in ppb) were available in the same period from the National Air Pollution Surveillance (NAPS) network of Environment Canada. Daily level of pollutants was computed as the 24-hour mean based on hourly measurements in different stations, and then averaged across stations with no missing data, with an average of 4 stations per city. In total, missing data amount for 2·00% and 0·97% of the mortality and temperature series, respectively. These data were used and described in previous publications.[2,3]

Chile. We collected data from the city of Santiago de Chile between 1st of January 2008 and 31st of December 2014. Daily mortality, obtained from Departamento de Estadísticas e Información de Salud (Ministerio de Salud), is represented by counts of deaths for all causes. Mean daily temperature (in ˚C), computed as 24-hour average based on hourly measurements, were obtained from Sistema de Información Nacional de Calidad del Aire (SINCA), Ministerio del Medio Ambiente. A single weather station (Parque Ohiggins) was selected. In total, missing data amount for 0·00% and 1·17% of the mortality and temperature series, respectively.

Czech Republic. We collected data from the 3 biggest cities (Prague, Brno, Ostrava) and a rural region (South Bohemia) between 1st January 1994 and 31st December 2015. Daily mortality is represented by counts of all-cause deaths (obtained from the Czech Statistical Office and the Institute of Health Information and Statistics). Meteorological data (temperature and relative humidity) over the same period were obtained from stations operated by the Czech Hydrometeorological Institute (measurements in standard climatic terms 7:00, 14:00 and 21:00 local time, and daily means). For Prague, Brno and Ostrava, data from airport stations in given cities were used. Climatic variables representative for the rural region of South Bohemia was calculated as average from 3 stations (Ceske Budejovice, Kostelni Myslova, Pribyslav). In total, missing data amount for 0·00% and 0·00% of the mortality and temperature series, respectively.

Finland. We collected data from the city of Helsinki between the 1st of January 1994 and 31st of December 2011. Daily mortality, obtained from Statistics Finland, is represented by counts of deaths for all causes. A dataset containing minimum, mean, and maximum daily temperatures was obtained from the Finnish Meteorological Institute. In this dataset, point measurements from the weather measuring stations around the country have been interpolated onto a 10×10 km grid covering the whole of Finland, using a Kriging model. The temperature variables in the Helsinki Temperature Time-series have been extracted from the GIS-database for KKJ-coordinates 6675470:2552920 (KKJ, Finnish National Coordinate System based on ED50). These are the coordinates for weather measuring station Kallion urheilukenttä of Helsinki Region Environmental Services Authority HSY. In total, missing data amount for 0·00% and 0·00% of the mortality and temperature series, respectively.

France. We collected data from 18 cities (see full list in Table S1 below) between 1st of January 2000 and 31st of December 2010. Daily mortality, obtained from the French National Institute of Health and Medical Research (CepiDC), is represented by counts of deaths for all causes. Mean daily temperature (in ˚C), computed as the average between daily minimum and maximum, were obtained from the Meteo France. A single weather station was selected for each city; for 15 out 18 locations the weather station was located at the nearest airport. Two cities (Lille and Lens) have the same temperature series from the same weather station. In total, missing data amount for 0·00% and 0·06% of the mortality and temperature series, respectively.

Ireland. We collected data in 6 regions that cover all the island of Ireland; four in the Republic of Ireland (ROI) (North East of the Republic of Ireland, South East of the Republic of Ireland, North West of the Republic of

Ireland, South West of the Republic of Ireland), and two in Northern Ireland (NI) (West of Northern Ireland, East of Northern Ireland), between 1st of January 1984 and 31st of December 2007. Daily mortality, obtained from the Irish Central Statistics Office, and Northern Ireland Social Research Agency for ROI and NI, is represented by counts of deaths for non-external causes only (ICD-9: 0-799; ICD-10: A00-R99). Mean daily temperature (in ˚C) and relative humidity (in %), computed as the 24-hour average based on hourly measurements, were obtained from the Met Eireann, and the United Kingdom Meteorological Office for ROI and NI. Data from 10 weather station were used to build regional series for ROI, with at least two weather stations for each of the four ROI regions; while data from two weather stations were averaged to build regional series for the two NI regions. In total, missing data amount for 0·01% and 0·00% of the mortality and temperature series, respectively.

Mexico. We collected data from 10 metropolitan areas (see full list in Table S1 below) between 1st of January 1998 and 31st of December 2014. Daily mortality, obtained from the National Institute of Statistics, Geography and Informatics is represented by counts of deaths for all causes. Temperature and relative humidity data, were obtained from Servicio Meteorológico Nacional (SMN) and the Instituto Nacional de Ecología y Cambio Climático (INECC). Daily mean temperature as well as the maximum or minimum daily temperature was calculated using hourly data, with a minimum of adequacy of information of 50%. In the case of the data from airports, these were daily averages of temperature and relative humidity. We obtained the maximum and minimum temperatures as well as the average relative humidity across all stations that met the sufficiency criteria of having at least 75% data for the day. Measures of ozone (O_3, in ppb), particles (PM10, in $\mu g/m^3$), and fine particles measures ($PM_{2\cdot5}$, in $\mu g/m^3$) were available in the same period. Daily level of pollutants was computed as the 24-hour mean based on hourly measurements. In total, missing data account for 0·00% and 26·67% of the mortality and temperature series, respectively.

Moldova. We collected data from the city of Anenii Noi (1st of January 2003 to 31st of December 2010), Cahul (1st of January 2003 to 31st of December 2010), Chisinau (1st of January 2001 to 31st of December 2010), and Falesti (1st of January 2003 to 31st of December 2010). Daily mortality, obtained from National Centre for Health Management, Moldova, is represented by counts of deaths for all causes. Mean daily temperature (in ˚C) computed as the average between daily minimum and maximum, were obtained from State Hydrometeorological Service, Moldova. A single weather station was selected for each city. In total, missing data amount for 0·00% and 0·00% of the mortality and temperature series, respectively.

Philippines. We collected data from four cities (Cebu, Davao, Manila, and Quezon) between the 1st of January 2006 and 31st of December 2010. Daily mortality, obtained from Philippine Statistics Authority-National Statistics Office, is represented by counts of deaths for all causes. Mean daily temperature (in ˚C), and relative humidity (in %), computed as the 24-hour average based on hourly measurements, were obtained from Philippine Atmospheric Geophysical and Astronomical Services Administration, and National Oceanic and Atmospheric Administration. A single weather station was selected for each city. In particular, for Davao and Cebu we used the airport monitoring station closest to the city centre, while the weather station was located in Manila Port for Manila, and in the Science Garden for Quezon. In total, missing data amount for 0·14% and 0·01% of the mortality and temperature series, respectively.

Spain. We collected data from the 52 provincial capital cities (between 1st of January 1990 and 31st of December 2014. Daily mortality, obtained from the Spanish National Institute of Statistics (reference PB242/2016), is represented by counts of deaths for non-external causes only (ICD-9: 0-799; ICD-10: A00-R99). Mean daily temperature (in ℃), computed as the 24-hour average based on hourly measurements from Spain National Meteorology Agency stations were obtained through the European Climate Assessment & Dataset (available at http://www.ecad.eu/). A single weather station, located within the urban area or at the nearest airport, was selected for each city. Single-day missing values were imputed as the average of the days before and after. For periods longer than two days no imputation was done. In total, missing data amount for 0·00% and 2·00% of the mortality and temperature series after imputation, respectively.

Switzerland. We collected data from 8 cities (Basel, Bern, Zurich, Geneva Lausanne, Luzern, Lugano, St Gallen) between 1st January 1995 to 31st December 2013. Lugano also includes the small municipalities around the main city of Lugano with similar altitude. Daily mortality, provided by the Federal Office of Statistics (Switzerland), is represented by counts of deaths for all causes, Daily data on several meteorological indicators were collected from the IDA web database (Federal Office of Meteorology and Climatology, MeteoSwiss). A single weather station located within or near the urban area was selected for each city. The meteorological indicators were: mean daily temperature (in ˚C) and relative humidity (in %), computed as the 24-hour average based on hourly measurements. Daily measurements of nitrogen dioxide (NO2, in ppb), particles (PM10, in ppb), and ozone (O3, in ppb), were provided by the Immissionsdatenbank Luft (IDB, Federal Office of the Environment, Bern, Switzerland) and computed as 24-h average for the former two and as 1h-maximum for the latter. In total, missing data amount for 0·00% and 0·00% of the mortality and temperature series, respectively.

UK. We collected data in 9 regions of England (North East, North West, Yorkshire & Humber, East, East Midlands, West Midlands, London, South East, and South West) and Wales between the 1st of January 1990 and 31st of August 2012. Daily mortality, obtained from the Office of National Statistics, is represented by counts of deaths for all causes and for non-external causes only (ICD-9: 0-799; ICD-10: A00-R99). Mean daily temperature (in ˚C) computed from the 24-h average of hourly measurements) were obtained from the British Atmospheric Data Centre. An average of 29 stations contributed data to each regional series, from a minimum of 7 in London to a maximum of 44 in Wales. In total, missing data amount for 0·00% and 0·00% of the mortality and temperature series, respectively. These data were used and described in previous publications.[4,5]

Vietnam. We collected data from the cities of Ho Chi Minh City (1st of January 2010 to 31st of December 2013), and Hue (1st of January 2009 to 31st of December 2013). Since 1992, a mortality data-collecting system based on the commune health center has been introduced in an official book named A6 [Vietnam Ministry of Health. Decision No 822/BYT.QD to issue mortality reporting book A6/YTCS. 1992]. Data from the A6 are collected at the commune health centre level and then forwarded to the provincial and central levels. In this study, daily mortality data from the A6 mortality reporting system, is represented by counts of deaths for all causes and for non-external causes only (ICD-9: 0-799; ICD-10: A00-R99). Mean daily temperature (in ˚C), and relative humidity (in %) computed as computed from the 24-h average of hourly measurements, were obtained from National Oceanic and Atmospheric Administration's (NOAA) National Climate Data Center (NCDC). A single weather station was selected for each city. In total, missing data amount for 0·00% and 0·45% of the mortality and temperature series, respectively.

2. Detailed information on the analytical framework

The data and the analytical framework are described below. Part of the statistical analysis is based on previously published methods, applied in a recent assessment on the mortality burden attributable to outdoor temperature using the same dataset.[1] Here we will focus on illustrating original extensions, while steps in common with the previous analysis are only briefly summarized, with references to published material for details.

Observed series. Historical data on weather and mortality have been collected through the Multi-Country Multi-city (MCC) Collaborative Research Network (http://mccstudy.lshtm.ac.uk/). At the time of the analysis, the dataset included information from 451 locations within 23 countries aggregated in 9 regions, in largely overlapping periods ranging from 1st of January 1984 to 31st December 2015 (Table 1 in the main text). The dataset is composed of observed daily time series for daily mean temperature T_{obs} and mortality counts D_{obs} for all causes or non-external causes only (International Classification of Diseases – ICD-9: 0-799; ICD-10: A00-R99), in addition to series of other weather variables. Location-specific meta-variables include weather indices derived from the series of T_{obs} (*e.g.*, average and range of annual, summer and winter temperature), climatological zones based the Köppen-Geiger classification,[6] and country-specific gross domestic product (GDP) per capita. The variable T_{obs} was measured as the average across the 24h or between maximum and minimum daily temperature from a single or multiple monitoring stations within the administrative boundaries of each location, or in its proximity (*e.g.*, at the nearby airport).

Projected temperature series. We obtained climate projections in the form of daily temperature series for historical (1950-2005) and future (2006-2100) periods from a database developed in the Inter-Sectoral Impact Model Intercomparison Project (ISI-MIP, https://www.isimip.org/).[7] The ISI-MIP database includes single runs of General Circulation Models (GCMs) developed within the Coupled Model Intercomparison Project Phase 5 (CMIP5).[8] GCMs offer a representation of past, current, and projected climate consistent with scenarios of increases in radiative atmospheric forces, summarized by Representative Concentration Pathways (RCPs). Four different scenarios (RCP2·6, RCP4·5, RCP6·0, and RCP8·5), defined in the Fifth Assessment Report (AR5) of the United Nations Intergovernmental Panel on Climate Change (IPCC),[9] correspond to increasing concentration trajectories of green-house gases (GHGs) and related changes in climate. Specifically, the ISI-MIP Fast Track database provides temperature series for each RCP scenario of five GCMs, namely GFDL-ESM2M,[10,11] HadGEM2-ES,[12] IPSL-CM5A-LR,[13] MIROC-ESM-CHEM,[14] and NorESM1-M.[15,16] These five GCMs were shown to be representative of the range of projections of future climate across the CMIP5 models.[7] The model outputs were bias-corrected and downscaled through bi-linear interpolation at a 0·5°×0·5° spatial resolution and linear interpolated by day of the year.[17] The modelled daily temperature series T_{mod} for each of the 451 locations in the period 1990-2099 were extracted by linking the coordinates with the corresponding cells of the grid.

Projected mortality series. We computed the impact of non-optimal temperature under the assumption of stable populations, thus ignoring demographic changes. For this reason, the projected series of mortality counts D_{mod} was computed as the average for each day of the year from D_{obs}, and then repeated along the same projection period of T_{mod}.

Calibration. The five climate models (GCMs) described above are expected to reproduce the observed temperature distribution during the observed period with reasonable accuracy. However, differences between the T_{obs} and T_{mod} series can occur for several reasons, including different resolution of the data (point sources versus gridded) and poor performance of climate models in areas with sparse information from meteorological stations.[18,19] Non-negligible deviations between the two sources can produce biased results in the impact projections, when the T_{mod} series are applied to exposure-response relationships estimated using T_{obs}. For this reason, we additionally corrected T_{mod} using the bias-correction method that was developed and applied in ISI-MIP.[17] This approach produced T^*_{mod}, a series recalibrated using the monthly mean and the daily variability around the monthly mean of T_{obs}. This calibration method ensures that the trend (*i.e.*, the warming signal) in the original data is preserved. The accuracy of the calibration was assessed by visually comparing the cumulative distribution functions of T_{obs} and T^*_{mod} for each of the 451 locations, and it was found to be very high.

Estimating exposure-response relationships. We estimated the non-linear and lagged exposure-response between outdoor temperature T_{obs} and mortality D_{obs} in each location using a time series regression model with quasi-Poisson family:[20]

$$\log[E(D_{obs})] = \alpha + s(T_{obs};\boldsymbol{\theta}) + f(t;\boldsymbol{\beta}) + I(w;\boldsymbol{\gamma})$$

The bi-dimensional spline function s and coefficients $\boldsymbol{\theta}$ define an exposure-lag-response risk surface for temperature accounting for 21 days of lag.[21] These were then transformed to s^* and $\boldsymbol{\theta}^*$, representing uni-dimensional overall cumulative (net) exposure-response curves with reduced lag dimension.[22] We controlled for seasonal and long term trends with a natural cubic spline function f and for differences due to day of the week with an indicator function I. Details of model specification are provided in a previous article.[1] In this analysis,

we replaced the quadratic B-spline applied in the bi-dimensional function s to model the temperature dimension with a natural cubic B-spline with the same knots. This choice allows a linear extrapolation of the functions beyond the boundaries of T_{obs}, a step needed to project the risk using T^*_{mod} in a period characterized by a warming climate. Location-specific overall cumulative exposure-response curves obtained using the two functions were compared and did not reveal important differences. The exposure-response curves were represented as relative risk (RR), interpreted as the relative increase in risk of mortality associated with a certain temperature versus a reference value. The reference, chosen as the temperature corresponding to minimum mortality risk (T_{mm}), is interpreted as the optimal temperature for that specific location.

Pooling the information across locations. We then performed a meta-regression of the reduced coefficients $\boldsymbol{\theta}^*$,[22,23] following a model previously presented.[1] To partly account for the heterogeneity in the relationship, we included region, climate type (first letter of the Koppen-Geiger classification),[6], GDP, and average and range of mean daily temperature across the whole period as meta-predictor. The residual heterogeneity was relatively low, with an I^2 index of 39·0%,[24] similar to the original analysis including country indicators.[1] This change allows a stable estimation also in countries with a limited number of locations, now included in this extended analysis, and the partial characterization of within-country heterogeneity due to climatic and socio-economic conditions. We used the estimates from the meta-analytical model to derive the best linear unbiased prediction (BLUP) $\boldsymbol{\theta}^*_b$ of the coefficients in each location. This method allows areas with small daily mortality counts or short series to borrow information from larger populations that share similar characteristics[23]

Projecting attributable mortality. For each day of the series of each location, we computed the number of deaths D_{attr} attributed to non-optimal temperature as:

$$D_{attr} = D^*_{mod} \cdot \left(1 - e^{-\left(s^*(T^*_{mod};\boldsymbol{\theta}^*_b) - s^*(T_{mm};\boldsymbol{\theta}^*_b)\right)}\right)$$

This daily attributable mortality was then aggregated within periods and geographical regions, and the corresponding attributable fraction was computed as the ratio with the corresponding total number of deaths. We interpret this quantity as the excess mortality related to exposure to non-optimal temperature. We also separated components due to heat and cold by summing the subsets corresponding to days with temperatures higher or lower than T_{mm}.[1,25] It is important to note that the method used here applies exposure-response relationships estimated using historical data to future modelled temperature series, and then computes the number of attributable deaths using a baseline mortality averaged from the observed series. Therefore, the projected temperature-related impact must be interpreted under a scenario where no adaptation (i.e., modification of the exposure response function) or population changes (i.e., modification of the baseline mortality) occur. While previous studies have reported an attenuation in the risk association with heat in several population during the last decades,[26,27] findings on cold-related mortality are less clear,[26] and the current evidence and analytical methods do not provide a well-grounded approach to quantify adaptation to future climates in terms of changes in flexible temperature-mortality relationships. Furthermore, by not considering population changes, in terms of both increase, ageing and variation in mortality rates, we isolate impacts solely related to climate change from other determinants. The figures reported in this article should be therefore interpreted as potential impacts in a well-defined scenario, and not as predictions of future excess mortality.

Quantification of uncertainty. The main sources of uncertainty in the attributable mortality D_{attr} are related to the estimate of the exposure-response relationships and the variability in temperature projections. These quantities are represented by the covariance matrix $V(\boldsymbol{\theta}^*_b)$ of the reduced model coefficients and the variability of the five series T^*_{mod} generated in each GCM, respectively. We quantified this uncertainty by generating 1000 samples of the reduced BLUP coefficients through Monte Carlo simulations,[1,25] assuming a multivariate normal distribution for the estimated spline model coefficients, and then generating results for each of the five GCMs. We reported the results as point estimates, using the average across climate models (GCM-ensemble) obtained by the estimated coefficients, and as empirical confidence intervals, defined as the 2·5th and 97·5th percentiles of the empirical distribution across coefficients samples and GCMs. These empirical 95% confidence intervals (eCI) account for both sources of uncertainty. A quantitative comparison of the two components is provided by the ratio between the average standard deviation of the empirical distribution within each GCM, and the standard deviation of the average value between GCMs.

3. Illustration of the modelling procedure in two cities

Figure S1 illustrates the procedure to estimate temperature-related excess mortality in two cities located in temperate and sub-tropical areas, respectively, using a specific GMC/RCP combination. London, UK (left column), experiences an average increase of 3·4°C in T^*_{mod} from 2010-19 to 2090-99. This increases the number of days with temperatures higher than the optimal value (minimum mortality temperature T_{mm}, reported as MMT in the main text), and produces temperatures exceeding those experienced in the current period. Heat-related excess mortality rises accordingly from 0·4% to 2·7% of total deaths. However, the excess mortality due to cold, despite the decrease from 9·0% to 5·8%, remain the larger temperature-related component. The impact of climate change is more intense in the second city, Manila, Philippines (right column). The excess mortality in this location during the current period is 1·3% and 2·4%, for heat and cold, respectively. Although this city is projected to experience a lower warming (2·7°C), the shift moves almost the entire temperature distribution to levels higher than the T_{mm}. This eliminates any impact associated with cold, while it raises substantially the heat-related excess mortality to 14·8%.

This comparison emphasises the differential health impact of global warming across locations with different characteristics, and demonstrate the various factors contributing to its quantification. In addition, the graph highlights the issues related with extrapolating the exposure-response curves beyond the observed temperature range. First, the extrapolation generates a large uncertainty, represented by the wide confidence intervals, which explains the imprecision in the measure of net impacts, especially in tropical regions with a substantial shift in temperature. Second, the log-linear assumption applied when extrapolating the spline function can underpredict potential non-linear dependencies with extremely high future temperatures.

***Figure S1*: Temperature and excess mortality in different climates**

The graphs represent the estimated exposure-response curves with 95%CI (top panels), with minimum mortality temperature (T_{mm}, continuous vertical line) used as reference, and the two portions identifying increased risks for cold and heat (blue and red, respectively). The dashed part of the curve represents the extrapolation beyond the maximum temperature observed in 2010-19 (dashed vertical line). The mid panels display the distribution of T^*_{mod} for the current period (2010-19, grey area) and at the end of the century (2090-99, green area), projected using a specific climate model (NorESM1−M) and scenario (RCP8·5). The bottom panels represent the related distribution of excess mortality, expressed as the excess deaths (%) related to non-optimal temperatures compared with T_{mm}.

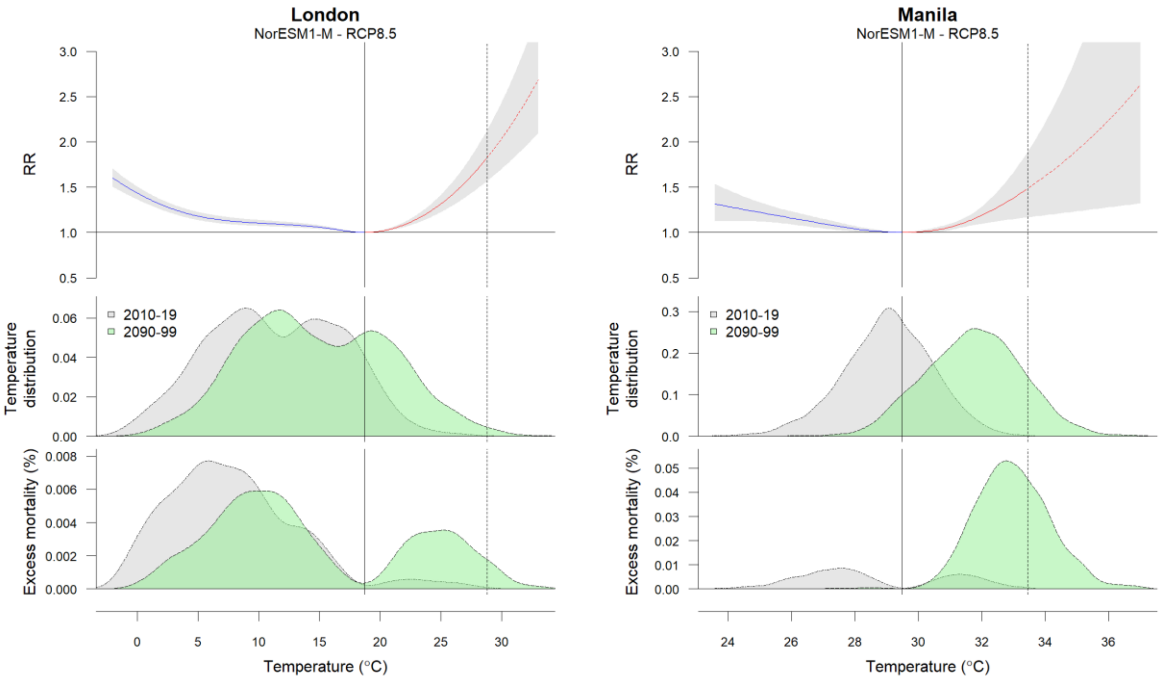

Table S1: **Heat-related, cold-related, and net change in excess mortality (%) by region, period and climate change scenario (95%eCI).**

		**RCP2·6**			**RCP4·5**			**RCP6·0**			**RCP8·5**		
		2010-19	2050-59	2090-99	2010-19	2050-59	2090-99	2010-19	2050-59	2090-99	2010-19	2050-59	2090-99
**North America**	Heat	0·5 (0·0 to 1·0)	0·8 (0·0 to 1·6)	0·7 (0·0 to 1·6)	0·5 (0·1 to 1·0)	1·1 (-0·0 to 2·4)	1·4 (-0·0 to 3·0)	0·5 (0·0 to 0·9)	0·9 (-0·0 to 1·8)	1·8 (-0·1 to 3·9)	0·5 (0·0 to 1·0)	1·5 (-0·1 to 3·1)	3·6 (-0·5 to 7·6)
	Cold	5·2 (2·6 to 7·7)	4·8 (2·3 to 7·2)	4·9 (2·4 to 7·3)	5·3 (2·6 to 7·7)	4·6 (2·2 to 6·9)	4·4 (2·0 to 6·7)	5·3 (2·6 to 7·8)	4·8 (2·3 to 7·1)	4·1 (1·9 to 6·4)	5·2 (2·5 to 7·7)	4·4 (2·1 to 6·6)	3·5 (1·6 to 5·5)
	Net	-	-0·1 (-0·5 to +0·2)	-0·1 (-0·4 to +0·3)	-	-0·1 (-0·8 to +0·6)	-0·0 (-1·1 to +0·9)	-	-0·1 (-0·8 to +0·4)	+0·1 (-1·4 to +1·7)	-	+0·1 (-1·0 to +1·2)	+1·4 (-2·2 to +4·7)
**Central America**	Heat	0·6 (0·1 to 1·1)	1·0 (0·2 to 1·7)	0·9 (0·2 to 1·6)	0·6 (0·1 to 1·1)	1·6 (0·3 to 3·1)	2·3 (0·4 to 4·9)	0·7 (0·1 to 1·5)	1·4 (0·2 to 3·1)	3·2 (0·4 to 6·8)	0·6 (0·1 to 1·2)	2·3 (0·4 to 4·1)	7·0 (0·4 to 13·7)
	Cold	4·8 (2·8 to 6·8)	3·8 (2·0 to 5·8)	3·9 (2·0 to 6·0)	5·0 (3·0 to 7·1)	3·4 (1·6 to 5·4)	2·8 (1·2 to 4·6)	4·8 (2·7 to 6·9)	3·5 (1·8 to 5·2)	2·1 (0·9 to 3·6)	4·6 (2·5 to 6·6)	2·6 (1·2 to 4·4)	1·2 (0·3 to 3·0)
	Net	-	-0·5 (-1·6 to +0·1)	-0·6 (-1·6 to +0·1)	-	-0·6 (-2·0 to +0·4)	-0·5 (-2·4 to +1·3)	-	-0·6 (-1·6 to +0·6)	-0·2 (-2·6 to +2·7)	-	-0·2 (-1·9 to +1·3)	+3·0 (-3·0 to +9·3)
**South America**	Heat	0·9 (0·2 to 1·8)	1·6 (0·2 to 3·5)	1·6 (0·0 to 3·6)	0·9 (0·3 to 1·6)	2·1 (-0·3 to 4·8)	2·9 (-1·3 to 6·8)	1·0 (0·3 to 1·8)	1·9 (0·0 to 4·0)	3·5 (-2·5 to 8·9)	1·0 (0·3 to 1·8)	3·1 (-1·5 to 7·3)	7·7 (-14·0 to 22·1)
	Cold	3·0 (1·3 to 4·8)	2·6 (1·1 to 4·4)	2·5 (1·1 to 4·0)	3·0 (1·4 to 4·6)	2·1 (1·0 to 3·4)	1·8 (0·7 to 3·1)	3·0 (1·4 to 4·6)	2·2 (1·0 to 3·7)	1·6 (0·6 to 2·9)	3·0 (1·4 to 4·7)	1·8 (0·7 to 3·2)	1·0 (0·3 to 1·9)
	Net	-	+0·2 (-0·9 to +1·3)	+0·1 (-1·1 to +1·4)	-	+0·3 (-1·9 to +2·4)	+0·7 (-3·3 to +4·1)	-	+0·1 (-1·4 to +1·7)	+1·1 (-4·7 to +6·1)	-	+0·8 (-3·3 to +4·5)	+4·6 (-17·1 to +18·6)
**North Europe**	Heat	0·3 (0·1 to 0·6)	0·5 (0·1 to 1·1)	0·5 (0·1 to 1·0)	0·3 (0·1 to 0·6)	0·8 (0·2 to 1·8)	0·9 (0·2 to 1·9)	0·3 (0·1 to 0·7)	0·7 (0·1 to 1·5)	1·4 (0·4 to 3·0)	0·3 (0·1 to 0·5)	1·0 (0·2 to 2·1)	2·5 (0·5 to 5·0)
	Cold	7·1 (4·9 to 9·3)	6·6 (4·3 to 8·9)	7·0 (4·7 to 9·2)	7·3 (5·2 to 9·5)	6·2 (4·1 to 8·3)	6·0 (3·8 to 8·5)	7·3 (5·0 to 9·5)	6·4 (4·3 to 8·5)	5·5 (3·5 to 7·5)	7·4 (5·2 to 9·6)	5·9 (3·8 to 8·0)	4·6 (2·6 to 7·0)
	Net	-	-0·3 (-0·8 to +0·0)	-0·0 (-0·4 to +0·4)	-	-0·6 (-1·1 to -0·2)	-0·8 (-1·6 to -0·2)	-	-0·6 (-1·2 to -0·1)	-0·7 (-1·9 to +0·6)	-	-0·8 (-1·4 to +0·1)	-0·6 (-2·3 to +1·6)
**Central Europe**	Heat	1·2 (0·7 to 1·7)	1·7 (0·9 to 2·8)	1·6 (0·8 to 2·8)	1·1 (0·7 to 1·7)	2·5 (1·4 to 4·2)	3·0 (1·6 to 5·5)	1·1 (0·6 to 1·5)	1·8 (1·0 to 3·1)	4·0 (2·1 to 6·6)	1·1 (0·6 to 1·5)	3·2 (1·7 to 5·2)	7·1 (2·9 to 11·3)
	Cold	8·0 (4·9 to 11·0)	7·5 (4·4 to 10·6)	7·7 (4·6 to 10·6)	8·2 (5·0 to 11·2)	7·2 (4·2 to 10·0)	6·9 (4·0 to 10·0)	8·2 (5·0 to 11·2)	7·4 (4·3 to 10·3)	6·5 (3·8 to 9·4)	8·1 (5·0 to 11·2)	6·9 (4·0 to 9·7)	5·6 (3·0 to 8·6)
	Net	-	+0·0 (-0·3 to +0·3)	+0·2 (-0·2 to +0·6)	-	+0·4 (-0·3 to +1·5)	+0·6 (-0·2 to +2·4)	-	-0·0 (-0·7 to +0·7)	+1·3 (+0·1 to +3·4)	-	+0·8 (-0·1 to +2·4)	+3·5 (+0·4 to +7·1)
**South Europe**	Heat	1·8 (1·2 to 2·8)	2·8 (1·5 to 5·0)	2·4 (1·3 to 4·4)	1·6 (1·0 to 2·5)	3·5 (2·3 to 4·9)	4·6 (2·3 to 7·3)	1·6 (1·0 to 2·2)	2·8 (1·5 to 4·7)	5·8 (3·5 to 9·3)	1·6 (1·0 to 2·3)	4·8 (2·7 to 7·7)	10·5 (5·6 to 17·3)
	Cold	6·0 (2·9 to 9·1)	5·5 (2·4 to 8·5)	5·6 (2·5 to 8·6)	6·1 (3·0 to 9·2)	5·2 (2·3 to 8·1)	4·8 (1·9 to 7·7)	6·1 (3·0 to 9·1)	5·3 (2·3 to 8·2)	4·4 (1·8 to 7·2)	6·0 (2·9 to 9·1)	4·7 (1·9 to 7·5)	3·5 (1·1 to 6·3)
	Net	-	+0·4 (-0·2 to +1·5)	+0·2 (-0·6 to +1·0)	-	+1·0 (+0·4 to +2·2)	+1·6 (+0·1 to +3·9)	-	+0·3 (-0·6 to +1·6)	+2·6 (+0·9 to +5·2)	-	+1·8 (-0·0 to +3·9)	+6·4 (+2·3 to +12·3)
**East Asia**	Heat	0·5 (0·2 to 0·9)	0·9 (0·2 to 1·7)	0·8 (0·2 to 1·3)	0·4 (0·1 to 0·7)	1·0 (0·3 to 1·7)	1·3 (0·4 to 2·3)	0·4 (0·1 to 0·7)	0·8 (0·2 to 1·5)	1·8 (0·5 to 3·2)	0·5 (0·2 to 0·9)	1·3 (0·4 to 2·4)	3·2 (0·9 to 5·6)
	Cold	8·7 (5·7 to 11·4)	8·1 (5·2 to 10·8)	8·2 (5·3 to 10·9)	8·8 (5·9 to 11·6)	7·7 (4·9 to 10·3)	7·4 (4·6 to 10·0)	8·9 (6·0 to 11·7)	8·0 (5·2 to 10·6)	6·9 (4·3 to 9·5)	8·7 (5·7 to 11·4)	7·3 (4·5 to 9·8)	5·9 (3·4 to 8·5)
	Net	-	-0·3 (-0·8 to -0·0)	-0·2 (-0·7 to +0·2)	-	-0·5 (-1·0 to -0·0)	-0·6 (-1·4 to +0·1)	-	-0·6 (-1·0 to -0·3)	-0·7 (-1·7 to +0·4)	-	-0·6 (-1·5 to -0·1)	-0·1 (-2·1 to +1·6)
**South East Asia**	Heat	1·6 (0·5 to 3·6)	2·7 (0·7 to 5·5)	2·8 (0·7 to 5·3)	1·8 (0·5 to 3·6)	4·0 (0·9 to 7·3)	5·4 (1·0 to 10·1)	1·6 (0·5 to 3·2)	3·5 (0·9 to 7·3)	7·8 (0·8 to 16·1)	1·7 (0·5 to 3·1)	5·9 (0·9 to 10·7)	16·7 (-1·7 to 33·2)
	Cold	3·1 (1·6 to 4·5)	2·3 (1·0 to 4·5)	2·3 (1·1 to 3·7)	2·9 (1·6 to 4·3)	1·8 (0·9 to 3·0)	1·6 (0·6 to 3·2)	2·9 (1·6 to 4·3)	2·0 (0·9 to 3·4)	1·3 (0·4 to 2·7)	3·0 (1·6 to 4·3)	1·4 (0·6 to 2·9)	0·7 (0·1 to 1·7)
	Net	-	+0·3 (-0·7 to +1·3)	+0·4 (-0·7 to +1·7)	-	+1·1 (-0·9 to +2·9)	+2·3 (-1·0 to +6·0)	-	+1·0 (-0·7 to +3·5)	+4·5 (-1·4 to +11·6)	-	+2·7 (-1·3 to +6·4)	+12·7 (-4·7 to +28·1)
**Australia**	Heat	0·5 (0·1 to 0·9)	0·7 (0·1 to 1·3)	0·8 (0·1 to 1·5)	0·5 (0·1 to 1·0)	0·9 (0·1 to 1·7)	1·2 (0·2 to 2·2)	0·5 (0·1 to 1·0)	0·8 (0·1 to 1·6)	1·5 (0·3 to 2·9)	0·5 (0·1 to 1·0)	1·2 (0·2 to 2·5)	3·2 (0·6 to 6·3)
	Cold	7·5 (3·5 to 11·0)	6·9 (3·1 to 10·3)	7·1 (3·3 to 10·6)	7·6 (3·6 to 11·1)	6·4 (2·7 to 9·8)	5·9 (2·4 to 9·2)	7·5 (3·6 to 11·0)	6·5 (2·8 to 9·9)	5·3 (2·0 to 8·4)	7·6 (3·6 to 11·0)	5·7 (2·3 to 8·9)	3·7 (1·0 to 6·4)
	Net	-	-0·3 (-0·9 to +0·1)	-0·1 (-0·4 to +0·3)	-	-0·8 (-1·6 to -0·2)	-1·0 (-2·0 to -0·1)	-	-0·7 (-1·4 to -0·2)	-1·2 (-2·6 to -0·1)	-	-1·1 (-2·2 to -0·2)	-1·2 (-3·6 to +1·4)

Table S2: **Heat-related, cold-related, and net excess mortality (%) by country, period and climate change scenario (95%eCI).**

		RCP2·6			RCP4·5			RCP6·0			RCP8·5		
		2010-19	2050-59	2090-99	2010-19	2050-59	2090-99	2010-19	2050-59	2090-99	2010-19	2050-59	2090-99
**Australia**	Heat	0·5 (0·1 to 0·9)	0·7 (0·1 to 1·3)	0·8 (0·1 to 1·5)	0·5 (0·1 to 1·0)	0·9 (0·1 to 1·7)	1·2 (0·2 to 2·2)	0·5 (0·1 to 1·0)	0·8 (0·1 to 1·6)	1·5 (0·3 to 2·9)	0·5 (0·1 to 1·0)	1·2 (0·2 to 2·5)	3·2 (0·6 to 6·3)
	Cold	7·5 (3·5 to 11·0)	6·9 (3·1 to 10·3)	7·1 (3·3 to 10·6)	7·6 (3·6 to 11·1)	6·4 (2·7 to 9·8)	5·9 (2·4 to 9·2)	7·5 (3·6 to 11·0)	6·5 (2·8 to 9·9)	5·3 (2·0 to 8·4)	7·6 (3·6 to 11·0)	5·7 (2·3 to 8·9)	3·7 (1·0 to 6·4)
	Net	-	-0·3 (-0·9 to +0·1)	-0·1 (-0·4 to +0·3)	-	-0·8 (-1·6 to -0·2)	-1·0 (-2·0 to -0·1)	-	-0·7 (-1·4 to -0·2)	-1·2 (-2·6 to -0·1)	-	-1·1 (-2·2 to -0·2)	-1·2 (-3·6 to +1·4)
**Brazil**	Heat	1·0 (0·2 to 2·0)	1·7 (0·1 to 3·9)	1·7 (-0·0 to 4·0)	1·0 (0·3 to 1·7)	2·3 (-0·5 to 5·2)	3·1 (-1·6 to 7·5)	1·1 (0·3 to 2·0)	2·0 (-0·1 to 4·4)	3·8 (-3·1 to 9·9)	1·1 (0·3 to 2·0)	3·3 (-1·9 to 8·0)	8·2 (-17·1 to 24·7)
	Cold	2·6 (1·1 to 4·2)	2·1 (0·9 to 4·0)	2·1 (0·9 to 3·5)	2·6 (1·2 to 3·9)	1·7 (0·8 to 2·7)	1·4 (0·6 to 2·6)	2·5 (1·2 to 3·9)	1·7 (0·8 to 3·1)	1·2 (0·5 to 2·2)	2·6 (1·2 to 4·0)	1·3 (0·5 to 2·6)	0·6 (0·2 to 1·3)
	Net	-	+0·3 (-1·0 to +1·6)	+0·2 (-1·2 to +1·7)	-	+0·4 (-2·1 to +2·8)	+0·9 (-3·8 to +4·8)	-	+0·2 (-1·5 to +2·0)	+1·4 (-5·4 to +7·1)	-	+1·0 (-3·8 to +5·2)	+5·1 (-20·1 to +21·2)
**Canada**	Heat	0·7 (0·2 to 1·2)	0·9 (0·2 to 1·8)	0·9 (0·2 to 1·7)	0·7 (0·2 to 1·3)	1·3 (0·4 to 2·6)	1·8 (0·4 to 3·7)	0·6 (0·2 to 1·0)	1·1 (0·3 to 1·9)	2·1 (0·6 to 3·8)	0·7 (0·2 to 1·2)	1·8 (0·6 to 3·0)	4·3 (1·2 to 7·9)
	Cold	6·2 (3·4 to 9·0)	5·9 (3·1 to 8·5)	5·9 (3·1 to 8·6)	6·3 (3·4 to 9·0)	5·7 (3·0 to 8·3)	5·4 (2·8 to 8·0)	6·4 (3·4 to 9·1)	5·8 (3·1 to 8·5)	5·2 (2·7 to 7·7)	6·2 (3·4 to 9·0)	5·5 (2·9 to 8·0)	4·5 (2·2 to 6·9)
	Net	-	-0·1 (-0·4 to +0·2)	-0·1 (-0·3 to +0·3)	-	+0·1 (-0·4 to +0·7)	+0·2 (-0·7 to +1·4)	-	-0·0 (-0·8 to +0·6)	+0·4 (-0·8 to +1·7)	-	+0·3 (-0·5 to +1·2)	+1·9 (-0·6 to +4·8)
**Chile**	Heat	0·6 (0·2 to 1·2)	0·9 (0·3 to 1·6)	0·9 (0·3 to 1·5)	0·7 (0·2 to 1·3)	1·4 (0·3 to 2·9)	1·8 (0·5 to 3·1)	0·7 (0·2 to 1·3)	1·2 (0·4 to 2·1)	2·2 (0·6 to 3·8)	0·8 (0·2 to 1·4)	2·0 (0·5 to 3·3)	4·8 (1·0 to 9·2)
	Cold	5·3 (2·2 to 8·3)	4·8 (1·9 to 7·7)	4·8 (1·8 to 7·6)	5·4 (2·3 to 8·4)	4·5 (1·7 to 7·3)	4·1 (1·4 to 6·8)	5·5 (2·4 to 8·6)	4·7 (1·8 to 7·5)	4·0 (1·4 to 6·6)	5·5 (2·4 to 8·5)	4·3 (1·6 to 6·9)	2·9 (0·7 to 5·3)
	Net	-	-0·2 (-0·7 to +0·2)	-0·3 (-0·7 to +0·0)	-	-0·2 (-1·0 to +0·8)	-0·2 (-1·3 to +0·8)	-	-0·3 (-1·0 to +0·3)	-0·1 (-1·3 to +1·3)	-	+0·0 (-1·0 to +1·1)	+1·5 (-2·0 to +5·4)
**China**	Heat	0·9 (-0·0 to 1·8)	1·4 (-0·0 to 3·0)	1·4 (-0·0 to 3·1)	0·8 (-0·0 to 1·8)	1·7 (0·0 to 3·3)	2·3 (0·0 to 4·6)	0·7 (-0·0 to 1·5)	1·4 (0·0 to 3·4)	3·1 (0·1 to 6·3)	1·0 (0·0 to 1·9)	2·4 (0·0 to 5·1)	6·1 (0·2 to 12·0)
	Cold	10·8 (6·8 to 14·3)	10·0 (6·2 to 13·6)	10·1 (6·3 to 13·5)	10·9 (6·9 to 14·5)	9·6 (5·9 to 12·9)	9·1 (5·6 to 12·4)	11·0 (7·0 to 14·5)	10·0 (6·2 to 13·4)	8·6 (5·2 to 12·0)	10·8 (6·8 to 14·3)	8·9 (5·3 to 12·4)	7·1 (4·0 to 10·6)
	Net	-	-0·3 (-1·1 to +0·2)	-0·2 (-0·8 to +0·4)	-	-0·5 (-1·4 to +0·6)	-0·3 (-1·9 to +1·0)	-	-0·3 (-1·2 to +0·4)	+0·0 (-2·3 to +2·2)	-	-0·4 (-2·1 to +0·9)	+1·5 (-3·4 to +5·9)
**Czech Republic**	Heat	0·9 (0·5 to 1·6)	1·2 (0·4 to 2·0)	1·4 (0·7 to 2·7)	0·8 (0·4 to 1·6)	1·9 (0·9 to 3·8)	2·3 (1·0 to 4·6)	0·8 (0·4 to 1·2)	1·5 (0·8 to 2·9)	3·0 (1·2 to 5·4)	0·8 (0·4 to 1·4)	2·3 (1·0 to 4·6)	5·1 (1·7 to 10·0)
	Cold	8·5 (5·4 to 11·3)	7·9 (4·9 to 10·9)	8·1 (5·1 to 10·9)	8·6 (5·5 to 11·5)	7·6 (4·7 to 10·4)	7·4 (4·6 to 10·3)	8·5 (5·5 to 11·4)	7·8 (4·9 to 10·7)	7·1 (4·3 to 9·8)	8·5 (5·5 to 11·5)	7·4 (4·6 to 10·2)	6·1 (3·6 to 8·9)
	Net	-	-0·2 (-0·6 to +0·2)	+0·1 (-0·4 to +0·7)	-	+0·1 (-0·6 to +1·4)	+0·3 (-0·7 to +1·8)	-	+0·0 (-0·6 to +1·0)	+0·7 (-0·2 to +2·5)	-	+0·3 (-0·5 to +1·9)	+1·9 (-0·5 to +5·9)
**Finland**	Heat	0·7 (0·1 to 1·3)	0·9 (0·1 to 1·8)	1·0 (0·2 to 1·9)	0·6 (0·1 to 1·1)	1·2 (0·2 to 2·3)	1·7 (0·2 to 3·3)	0·5 (0·1 to 0·9)	1·2 (0·2 to 2·4)	1·9 (0·3 to 3·7)	0·6 (0·1 to 1·2)	1·5 (0·3 to 2·8)	3·9 (0·7 to 7·2)
	Cold	7·2 (3·8 to 10·3)	6·6 (3·4 to 9·7)	6·8 (3·4 to 10·2)	7·5 (4·1 to 10·7)	6·3 (3·2 to 9·4)	6·0 (3·0 to 9·2)	7·6 (4·1 to 10·8)	6·4 (3·2 to 9·6)	5·5 (2·6 to 8·3)	7·4 (3·9 to 10·6)	5·8 (2·9 to 8·7)	4·6 (2·0 to 7·4)
	Net	-	-0·3 (-0·8 to +0·2)	-0·1 (-0·6 to +0·5)	-	-0·6 (-1·6 to +0·1)	-0·4 (-1·6 to +0·6)	-	-0·5 (-1·4 to +0·3)	-0·6 (-2·2 to +1·1)	-	-0·7 (-1·7 to +0·3)	+0·5 (-2·2 to +3·3)
**France**	Heat	1·2 (0·8 to 1·9)	1·8 (1·0 to 3·1)	1·7 (0·8 to 3·1)	1·2 (0·8 to 1·8)	2·7 (1·7 to 4·4)	3·3 (1·8 to 5·8)	1·2 (0·7 to 1·6)	1·9 (1·1 to 3·2)	4·4 (2·5 to 7·1)	1·1 (0·7 to 1·6)	3·5 (2·0 to 5·5)	7·7 (3·3 to 12·0)
	Cold	8·0 (4·9 to 11·0)	7·5 (4·5 to 10·5)	7·7 (4·7 to 10·6)	8·2 (5·1 to 11·1)	7·1 (4·3 to 9·9)	6·9 (4·0 to 10·0)	8·2 (5·1 to 11·1)	7·3 (4·4 to 10·3)	6·5 (3·8 to 9·2)	8·2 (5·1 to 11·1)	6·8 (4·0 to 9·6)	5·6 (3·1 to 8·6)
	Net	-	+0·1 (-0·3 to +0·4)	+0·2 (-0·2 to +0·7)	-	+0·5 (-0·2 to +1·5)	+0·8 (-0·1 to +2·5)	-	-0·1 (-0·8 to +0·6)	+1·5 (+0·1 to +3·7)	-	+1·0 (-0·1 to +2·5)	+4·0 (+0·6 to +7·7)
**Ireland**	Heat	0·1 (-0·2 to 0·4)	0·2 (-0·3 to 0·7)	0·2 (-0·2 to 0·7)	0·1 (-0·2 to 0·5)	0·3 (-0·4 to 1·2)	0·4 (-0·5 to 1·3)	0·1 (-0·2 to 0·5)	0·3 (-0·4 to 1·1)	0·6 (-0·7 to 2·1)	0·1 (-0·2 to 0·4)	0·4 (-0·6 to 1·4)	1·1 (-1·5 to 4·2)
	Cold	8·3 (5·2 to 11·2)	7·7 (4·6 to 10·7)	8·1 (5·0 to 11·1)	8·6 (5·4 to 11·6)	7·3 (4·3 to 10·2)	7·0 (4·0 to 10·4)	8·5 (5·3 to 11·5)	7·4 (4·4 to 10·3)	6·4 (3·7 to 9·1)	8·6 (5·4 to 11·7)	6·9 (4·0 to 9·8)	5·3 (2·7 to 8·6)
	Net	-	-0·6 (-1·3 to -0·0)	-0·2 (-0·9 to +0·4)	-	-1·1 (-2·2 to -0·6)	-1·3 (-2·4 to -0·3)	-	-1·0 (-1·6 to -0·2)	-1·6 (-3·3 to -0·5)	-	-1·4 (-2·3 to -0·6)	-2·3 (-5·2 to +0·4)

**Italy**	Heat	1·8 (1·1 to 2·7)	2·8 (1·4 to 4·4)	2·6 (1·5 to 4·3)	1·5 (0·9 to 2·2)	3·5 (2·3 to 5·2)	4·6 (2·4 to 7·8)	1·6 (0·9 to 2·3)	2·9 (1·7 to 4·4)	5·9 (3·3 to 8·6)	1·6 (1·0 to 2·5)	4·7 (2·5 to 7·0)	10·0 (5·0 to 14·3)
	Cold	6·7 (3·6 to 9·7)	6·1 (3·0 to 9·3)	6·2 (3·2 to 9·2)	6·8 (3·7 to 9·9)	5·7 (2·8 to 8·6)	5·4 (2·5 to 8·5)	6·8 (3·7 to 9·8)	6·0 (3·0 to 9·0)	5·1 (2·4 to 7·9)	6·7 (3·6 to 9·7)	5·3 (2·5 to 8·2)	4·1 (1·5 to 7·1)
	Net	-	+0·3 (-0·1 to +0·9)	+0·3 (-0·4 to +0·8)	-	+0·9 (+0·2 to +2·4)	+1·7 (+0·3 to +4·6)	-	+0·4 (-0·6 to +1·2)	+2·5 (+1·0 to +4·4)	-	+1·7 (+0·1 to +3·4)	+5·7 (+1·9 to +9·3)
**Japan**	Heat	0·4 (0·2 to 0·6)	0·7 (0·2 to 1·4)	0·6 (0·3 to 0·9)	0·3 (0·1 to 0·5)	0·8 (0·3 to 1·3)	1·0 (0·3 to 1·8)	0·3 (0·1 to 0·6)	0·6 (0·3 to 1·0)	1·4 (0·6 to 2·6)	0·4 (0·2 to 0·7)	1·0 (0·4 to 1·7)	2·4 (0·9 to 3·9)
	Cold	8·1 (5·3 to 10·7)	7·5 (4·8 to 10·1)	7·7 (5·0 to 10·2)	8·3 (5·5 to 10·8)	7·2 (4·6 to 9·7)	6·9 (4·3 to 9·4)	8·4 (5·6 to 11·0)	7·4 (4·8 to 9·9)	6·4 (4·0 to 8·9)	8·1 (5·4 to 10·7)	6·8 (4·2 to 9·2)	5·5 (3·2 to 7·9)
	Net	-	-0·3 (-0·8 to -0·1)	-0·3 (-0·8 to +0·2)	-	-0·6 (-0·9 to -0·2)	-0·7 (-1·4 to -0·2)	-	-0·7 (-1·1 to -0·4)	-0·9 (-1·8 to -0·1)	-	-0·7 (-1·5 to -0·3)	-0·7 (-1·9 to +0·4)
**Mexico**	Heat	1·3 (0·4 to 3·3)	2·9 (0·7 to 8·1)	2·4 (0·9 to 4·8)	1·7 (0·5 to 4·5)	4·4 (1·5 to 8·8)	6·1 (2·1 to 11·0)	1·2 (0·4 to 2·1)	3·7 (1·3 to 7·6)	9·7 (2·5 to 20·1)	1·6 (0·6 to 3·0)	6·4 (2·2 to 10·9)	22·0 (3·8 to 39·4)
	Cold	2·9 (1·3 to 4·5)	1·8 (0·6 to 3·6)	1·9 (0·8 to 3·2)	2·8 (1·3 to 4·4)	1·3 (0·4 to 2·3)	1·0 (0·3 to 2·0)	3·0 (1·4 to 4·7)	1·4 (0·4 to 2·9)	0·7 (0·1 to 1·8)	2·6 (1·1 to 4·2)	0·8 (0·2 to 1·9)	0·2 (0·0 to 0·7)
	Net	-	+0·4 (-0·9 to +3·1)	+0·1 (-0·9 to +1·2)	-	+1·2 (-1·0 to +3·7)	+2·6 (-0·8 to +7·4)	-	+1·0 (-1·1 to +4·2)	+6·2 (-0·6 to +15·9)	-	+3·0 (-0·5 to +7·3)	+17·9 (+0·4 to +34·5)
**Moldova**	Heat	0·5 (0·1 to 0·9)	0·9 (0·3 to 2·1)	0·8 (0·2 to 1·6)	0·4 (0·1 to 0·8)	1·2 (0·4 to 2·2)	1·4 (0·4 to 2·7)	0·4 (0·1 to 0·7)	0·8 (0·2 to 1·3)	2·0 (0·6 to 3·9)	0·5 (0·1 to 0·9)	1·4 (0·5 to 2·4)	3·5 (1·2 to 6·1)
	Cold	8·7 (5·8 to 11·5)	8·2 (5·5 to 11·0)	8·3 (5·5 to 11·0)	8·8 (5·9 to 11·6)	8·0 (5·3 to 10·6)	7·7 (5·0 to 10·3)	8·9 (5·9 to 11·7)	8·2 (5·4 to 10·9)	7·3 (4·8 to 9·9)	8·7 (5·8 to 11·5)	7·6 (5·0 to 10·2)	6·5 (4·2 to 9·0)
	Net	-	+0·0 (-0·4 to +0·6)	-0·0 (-0·3 to +0·3)	-	-0·1 (-0·6 to +0·5)	-0·1 (-0·8 to +0·7)	-	-0·4 (-0·7 to -0·1)	-0·1 (-1·1 to +1·6)	-	-0·2 (-0·9 to +0·4)	+0·8 (-1·0 to +3·0)
**Philippines**	Heat	0·6 (0·1 to 1·1)	1·0 (0·2 to 1·7)	0·9 (0·2 to 1·6)	0·6 (0·1 to 1·1)	1·6 (0·3 to 3·1)	2·3 (0·4 to 4·9)	0·7 (0·1 to 1·5)	1·4 (0·2 to 3·1)	3·2 (0·4 to 6·8)	0·6 (0·1 to 1·2)	2·3 (0·4 to 4·1)	7·0 (0·4 to 13·7)
	Cold	4·8 (2·8 to 6·8)	3·8 (2·0 to 5·8)	3·9 (2·0 to 6·0)	5·0 (3·0 to 7·1)	3·4 (1·6 to 5·4)	2·8 (1·2 to 4·6)	4·8 (2·7 to 6·9)	3·5 (1·8 to 5·2)	2·1 (0·9 to 3·6)	4·6 (2·5 to 6·6)	2·6 (1·2 to 4·4)	1·2 (0·3 to 3·0)
	Net	-	-0·5 (-1·6 to +0·1)	-0·6 (-1·6 to +0·1)	-	-0·6 (-2·0 to +0·4)	-0·5 (-2·4 to +1·3)	-	-0·6 (-1·6 to +0·6)	-0·2 (-2·6 to +2·7)	-	-0·2 (-1·9 to +1·3)	+3·0 (-3·0 to +9·3)
**South Korea**	Heat	1·5 (0·7 to 2·2)	2·3 (1·1 to 3·8)	2·1 (1·2 to 3·1)	1·2 (0·6 to 1·7)	2·4 (1·0 to 3·7)	3·2 (1·4 to 5·1)	1·3 (0·7 to 2·1)	2·1 (1·2 to 3·3)	4·1 (1·6 to 8·2)	1·5 (0·8 to 2·3)	3·7 (2·1 to 6·0)	7·7 (3·7 to 11·6)
	Cold	11·1 (7·3 to 14·6)	10·4 (6·7 to 14·1)	10·6 (6·9 to 14·0)	11·6 (7·7 to 15·1)	10·3 (6·7 to 13·7)	10·1 (6·6 to 13·5)	11·2 (7·4 to 14·8)	10·4 (6·7 to 13·8)	9·6 (6·1 to 12·9)	11·2 (7·4 to 14·6)	9·8 (6·3 to 13·0)	8·3 (5·2 to 11·4)
	Net	-	+0·1 (-0·5 to +1·0)	+0·1 (-0·3 to +0·8)	-	-0·1 (-1·4 to +1·0)	+0·5 (-1·2 to +1·9)	-	-0·0 (-0·6 to +1·0)	+1·2 (-1·1 to +4·1)	-	+0·8 (-0·5 to +2·6)	+3·3 (+0·6 to +6·8)
**Spain**	Heat	1·8 (1·2 to 2·8)	2·8 (1·5 to 5·3)	2·4 (1·2 to 4·7)	1·6 (1·1 to 2·6)	3·5 (2·3 to 5·1)	4·6 (2·2 to 7·5)	1·6 (1·0 to 2·1)	2·7 (1·3 to 5·0)	5·8 (3·5 to 9·8)	1·6 (0·9 to 2·2)	4·8 (2·8 to 8·1)	10·8 (5·9 to 18·8)
	Cold	5·7 (2·6 to 8·8)	5·2 (2·2 to 8·3)	5·3 (2·2 to 8·3)	5·8 (2·7 to 8·9)	5·0 (2·1 to 7·9)	4·5 (1·7 to 7·3)	5·8 (2·6 to 8·8)	5·0 (2·0 to 7·9)	4·1 (1·5 to 6·9)	5·8 (2·6 to 8·9)	4·4 (1·6 to 7·1)	3·2 (0·9 to 5·9)
	Net	-	+0·5 (-0·2 to +1·8)	+0·1 (-0·7 to +1·2)	-	+1·1 (+0·4 to +2·1)	+1·6 (+0·0 to +3·7)	-	+0·3 (-0·9 to +1·9)	+2·6 (+0·9 to +5·7)	-	+1·9 (-0·1 to +4·4)	+6·7 (+2·4 to +13·8)
**Sweden**	Heat	0·4 (-0·2 to 1·0)	0·6 (-0·3 to 1·5)	0·6 (-0·3 to 1·6)	0·3 (-0·2 to 0·9)	0·7 (-0·3 to 1·8)	0·9 (-0·4 to 2·6)	0·3 (-0·2 to 0·8)	0·7 (-0·3 to 2·1)	1·2 (-0·5 to 3·0)	0·4 (-0·2 to 1·2)	0·9 (-0·4 to 2·4)	2·3 (-0·8 to 5·6)
	Cold	5·8 (2·3 to 9·1)	5·3 (2·0 to 8·5)	5·5 (2·1 to 8·8)	6·1 (2·5 to 9·4)	5·0 (1·8 to 8·2)	4·8 (1·6 to 8·0)	6·1 (2·5 to 9·4)	5·1 (1·9 to 8·3)	4·4 (1·5 to 7·3)	5·9 (2·4 to 9·3)	4·7 (1·6 to 7·7)	3·7 (1·1 to 6·6)
	Net	-	-0·3 (-0·9 to +0·1)	-0·1 (-0·7 to +0·3)	-	-0·7 (-1·7 to +0·0)	-0·7 (-2·1 to +0·3)	-	-0·6 (-1·6 to +0·3)	-0·8 (-2·5 to +0·8)	-	-0·8 (-1·8 to +0·3)	-0·4 (-3·1 to +2·3)
**Switzerland**	Heat	0·9 (0·3 to 1·5)	1·3 (0·5 to 2·5)	1·2 (0·4 to 2·3)	0·8 (0·3 to 1·4)	1·9 (0·7 to 3·9)	2·5 (0·8 to 5·8)	0·8 (0·3 to 1·4)	1·5 (0·5 to 2·9)	3·3 (1·1 to 6·4)	0·8 (0·3 to 1·4)	2·5 (0·8 to 5·1)	6·2 (1·8 to 12·4)
	Cold	5·5 (1·6 to 9·1)	5·1 (1·3 to 8·7)	5·2 (1·5 to 8·8)	5·6 (1·6 to 9·3)	4·9 (1·2 to 8·3)	4·6 (1·1 to 8·1)	5·6 (1·6 to 9·3)	5·0 (1·2 to 8·5)	4·4 (0·9 to 7·7)	5·5 (1·6 to 9·2)	4·6 (1·1 to 8·0)	3·7 (0·7 to 6·9)
	Net	-	+0·1 (-0·3 to +0·5)	+0·1 (-0·2 to +0·4)	-	+0·3 (-0·5 to +1·7)	+0·7 (-0·5 to +3·2)	-	-0·0 (-0·7 to +0·8)	+1·3 (-0·2 to +3·6)	-	+0·8 (-0·3 to +2·8)	+3·6 (+0·2 to +9·0)
**Taiwan**	Heat	1·3 (0·4 to 2·2)	1·8 (0·6 to 3·4)	1·9 (0·7 to 3·4)	1·3 (0·4 to 2·1)	2·3 (0·9 to 3·9)	3·0 (1·0 to 5·0)	1·2 (0·4 to 2·1)	2·1 (0·8 to 3·4)	3·8 (1·1 to 6·4)	1·3 (0·4 to 2·2)	3·2 (1·0 to 5·3)	7·3 (0·6 to 13·8)
	Cold	4·6 (2·9 to 6·4)	4·0 (2·3 to 5·9)	4·1 (2·5 to 5·9)	4·6 (2·9 to 6·3)	3·7 (2·2 to 5·3)	3·3 (1·8 to 5·1)	4·6 (2·9 to 6·4)	3·9 (2·3 to 5·5)	3·0 (1·6 to 4·6)	4·5 (2·8 to 6·3)	3·2 (1·7 to 4·8)	2·0 (0·7 to 3·7)
	Net	-	-0·0 (-0·9 to +0·6)	+0·2 (-0·5 to +0·9)	-	+0·2 (-0·7 to +1·0)	+0·5 (-1·1 to +1·9)	-	+0·1 (-0·7 to +0·8)	+1·0 (-1·3 to +3·1)	-	+0·6 (-1·1 to +2·1)	+3·5 (-2·7 to +9·4)
**Thailand**	Heat	1·8 (0·4 to 4·4)	2·8 (0·6 to 5·8)	3·1 (0·5 to 6·2)	2·1 (0·5 to 4·4)	4·4 (0·4 to 8·2)	5·8 (0·2 to 11·8)	1·9 (0·4 to 3·9)	3·8 (0·4 to 8·4)	8·1 (-0·6 to 18·3)	1·9 (0·4 to 3·6)	6·4 (-0·1 to 12·4)	16·6 (-6·4 to 35·8)
	Cold	2·6 (1·3 to 4·1)	1·9 (0·8 to 4·3)	1·8 (0·8 to 3·5)	2·4 (1·2 to 3·7)	1·4 (0·6 to 2·7)	1·3 (0·4 to 3·1)	2·4 (1·1 to 3·7)	1·7 (0·6 to 3·1)	1·0 (0·2 to 2·5)	2·5 (1·3 to 3·8)	1·1 (0·3 to 2·7)	0·5 (0·0 to 1·5)
	Net	-	+0·3 (-0·8 to +1·6)	+0·5 (-0·8 to +2·4)	-	+1·4 (-1·1 to +3·5)	+2·7 (-1·5 to +7·5)	-	+1·2 (-0·8 to +3·9)	+4·8 (-2·4 to +13·3)	-	+3·1 (-2·1 to +7·7)	+12·6 (-9·0 to +30·7)

**UK**	Heat	0·3 (0·1 to 0·6)	0·5 (0·2 to 1·2)	0·5 (0·1 to 1·0)	0·3 (0·1 to 0·7)	0·9 (0·3 to 1·8)	0·9 (0·2 to 1·9)	0·3 (0·1 to 0·7)	0·7 (0·1 to 1·5)	1·5 (0·4 to 3·1)	0·3 (0·1 to 0·5)	1·1 (0·3 to 2·2)	2·6 (0·5 to 5·1)
	Cold	7·1 (4·9 to 9·2)	6·5 (4·3 to 8·8)	6·9 (4·7 to 9·1)	7·3 (5·2 to 9·3)	6·1 (4·1 to 8·2)	5·9 (3·9 to 8·4)	7·2 (5·1 to 9·3)	6·3 (4·3 to 8·4)	5·4 (3·5 to 7·4)	7·3 (5·2 to 9·5)	5·8 (3·8 to 7·9)	4·5 (2·6 to 6·9)
	Net	-	-0·3 (-0·7 to +0·1)	+0·0 (-0·4 to +0·4)	-	-0·6 (-1·0 to -0·1)	-0·7 (-1·5 to -0·2)	-	-0·5 (-1·2 to -0·1)	-0·6 (-1·8 to +0·7)	-	-0·7 (-1·3 to +0·2)	-0·5 (-2·1 to +1·7)
**USA**	Heat	0·5 (0·0 to 1·0)	0·8 (0·0 to 1·6)	0·7 (-0·0 to 1·6)	0·5 (0·0 to 1·0)	1·1 (-0·0 to 2·4)	1·3 (-0·1 to 2·9)	0·5 (0·0 to 0·9)	0·9 (-0·1 to 1·8)	1·7 (-0·2 to 3·9)	0·5 (0·0 to 1·0)	1·5 (-0·1 to 3·1)	3·5 (-0·7 to 7·6)
	Cold	5·1 (2·5 to 7·5)	4·7 (2·2 to 7·0)	4·8 (2·3 to 7·1)	5·1 (2·5 to 7·6)	4·5 (2·1 to 6·8)	4·2 (2·0 to 6·5)	5·2 (2·5 to 7·7)	4·7 (2·2 to 6·9)	4·0 (1·8 to 6·2)	5·1 (2·4 to 7·5)	4·2 (2·0 to 6·4)	3·3 (1·5 to 5·3)
	Net	-	-0·1 (-0·5 to +0·2)	-0·1 (-0·4 to +0·2)	-	-0·1 (-0·8 to +0·6)	-0·1 (-1·1 to +0·9)	-	-0·2 (-0·8 to +0·4)	+0·1 (-1·5 to +1·7)	-	+0·1 (-1·0 to +1·2)	+1·3 (-2·4 to +4·7)
**Vietnam**	Heat	1·6 (0·8 to 2·5)	2·9 (1·4 to 4·7)	3·0 (1·3 to 5·0)	1·3 (0·6 to 2·0)	3·9 (1·8 to 6·7)	6·2 (2·1 to 10·7)	1·5 (0·5 to 4·0)	4·2 (1·5 to 8·6)	9·6 (3·9 to 16·3)	1·6 (0·6 to 3·4)	7·4 (3·1 to 12·2)	25·8 (8·7 to 45·2)
	Cold	3·2 (1·6 to 5·0)	2·2 (0·7 to 4·8)	2·3 (0·9 to 4·0)	3·3 (1·7 to 5·0)	1·6 (0·5 to 2·9)	1·2 (0·3 to 3·0)	3·4 (1·7 to 5·1)	1·9 (0·7 to 3·6)	0·9 (0·2 to 2·2)	3·4 (1·7 to 5·2)	1·1 (0·2 to 2·6)	0·3 (0·0 to 1·2)
	Net	-	+0·3 (-0·7 to +1·1)	+0·4 (-0·6 to +1·5)	-	+1·0 (-0·7 to +3·2)	+2·8 (-0·1 to +6·7)	-	+1·2 (-0·7 to +5·3)	+5·6 (+0·2 to +12·2)	-	+3·5 (-0·2 to +8·1)	+21·1 (+4·3 to +40·5)

***Figure S2*: Decadal temperature trends by region and scenario**

The graph shows the projected increase in temperature (°C, GCM-ensemble average), averaged by decade, region, and climate change scenario, compared to the current period (2010-19).

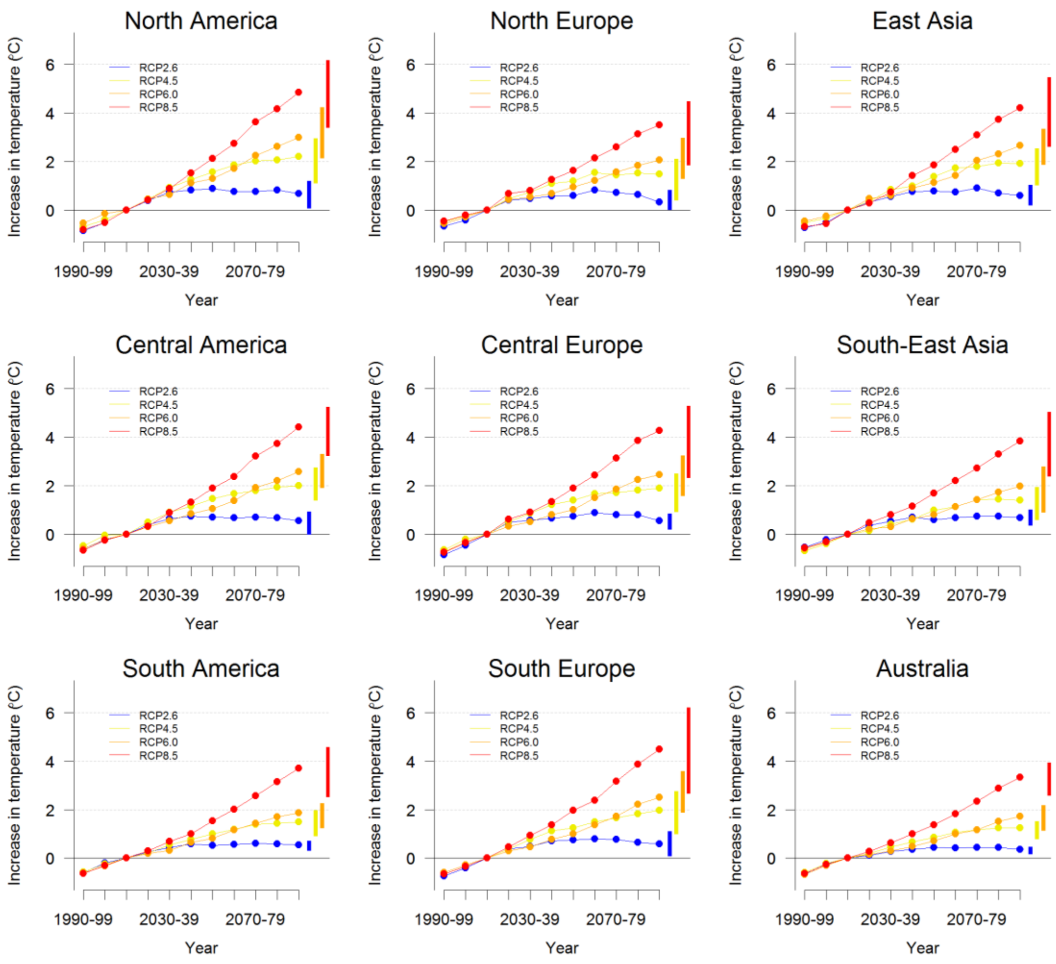

***Figure S3*: Map of location-specific changes in temperature**

The map shows the projected increase in temperature (°C, GCM-ensemble average) in 2090-99 compared to 2010-19 under RCP8·5 in the 451 locations.

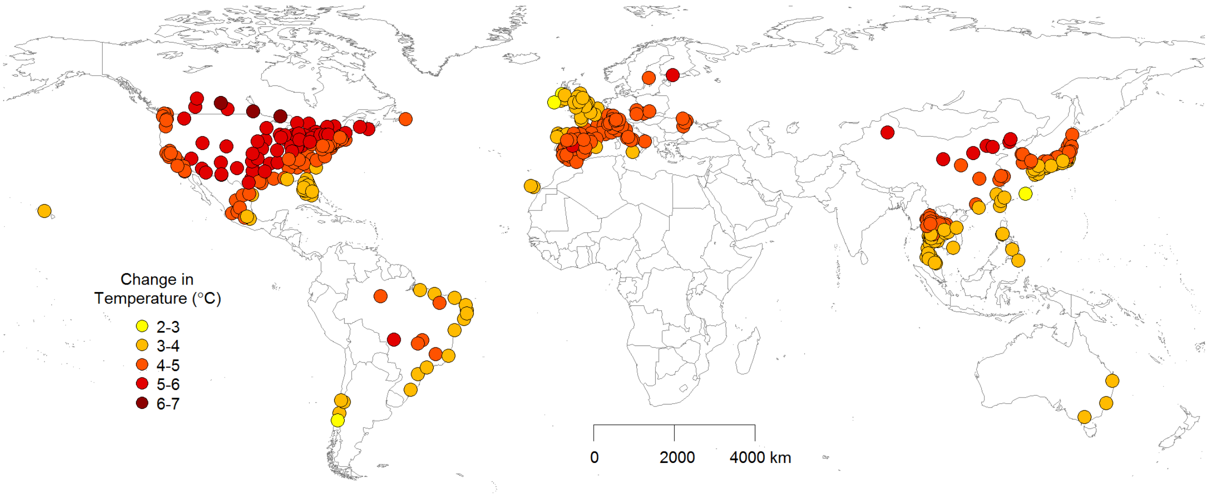

***Figure S4*: Maps of location-specific changes in temperature-related excess mortality**

The map shows the projected change in heat-related (top), cold-related (middle), and net (bottom) excess mortality (%) in 2090-99 compared to 2010-19 under RCP8·5 in the 451 locations.

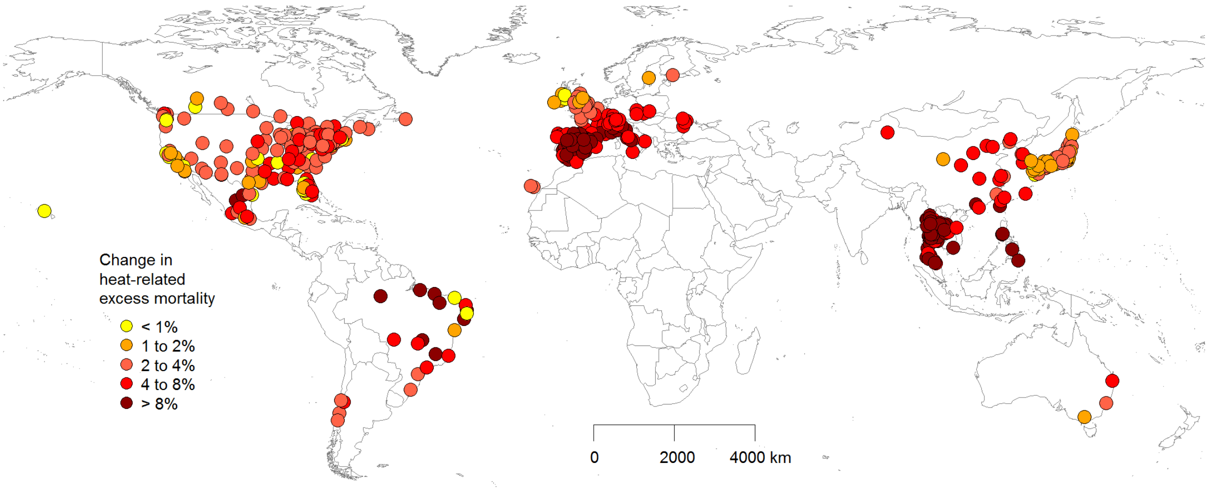

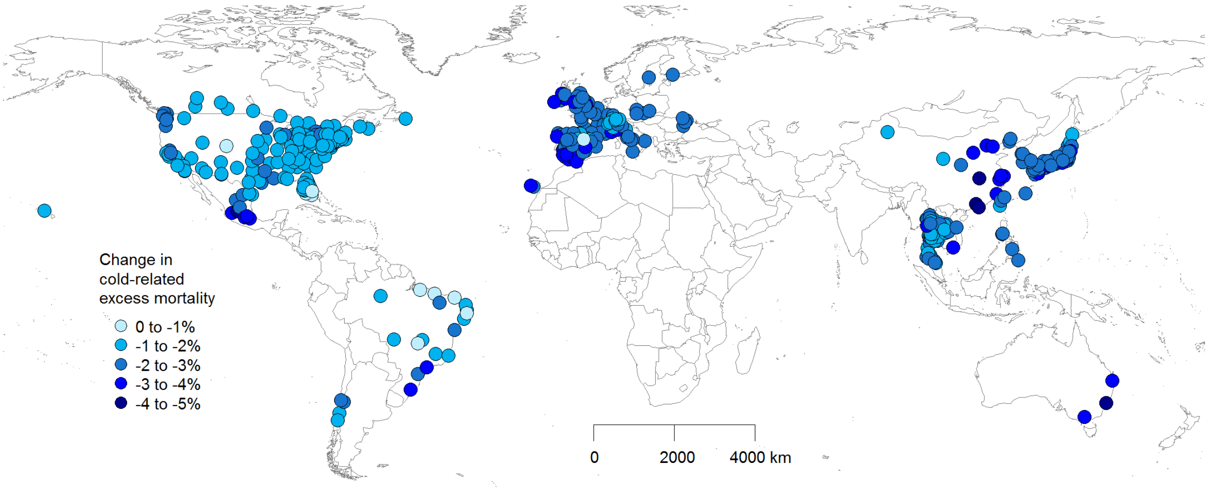

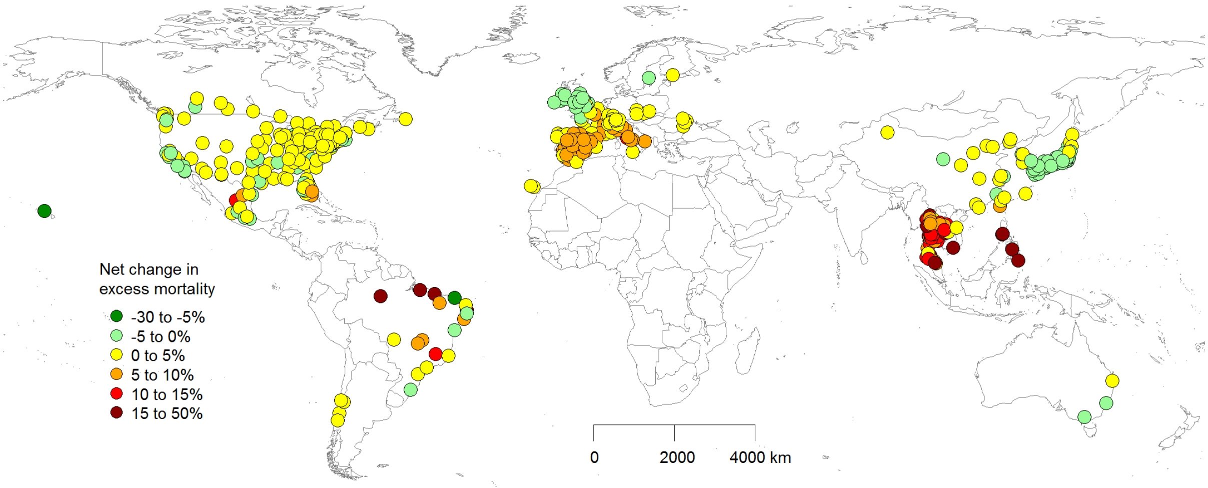

6. References

1. Gasparrini A, Guo Y, Hashizume M, et al. Mortality risk attributable to high and low ambient temperature: a multicountry observational study. *The Lancet* 2015; **386**(9991): 369-75.
2. Kaplan GG, Tanyingoh D, Dixon E, et al. Ambient ozone concentrations and the risk of perforated and nonperforated appendicitis: a multicity case-crossover study. *Environmental Health Perspectives* 2013; **121**(8): 939-43.
3. Martin SL, Cakmak S, Hebbern CA, Avramescu ML, Tremblay N. Climate change and future temperature-related mortality in 15 Canadian cities. *International Journal of Biometeorology* 2012; **56**(4): 605-19.
4. Armstrong BG, Chalabi Z, Fenn B, et al. The association of mortality with high temperatures in a temperate climate: England and Wales. *Journal of Epidemiology and Community Health* 2011; **65**(4): 340-5.
5. Gasparrini A, Armstrong B, Kovats S, Wilkinson P. The effect of high temperatures on cause-specific mortality in England and Wales. *Occupational and Environmental Medicine* 2012; **69**(1): 56-61.
6. Kottek M, Grieser J, Beck C, Rudolf B, Rubel F. World map of the Kooppen-Geiger climate classification updated. *Meteorologische Zeitschrift* 2006; **15**(3): 259-63.
7. Warszawski L, Frieler K, Huber V, Piontek F, Serdeczny O, Schewe J. The Inter-Sectoral Impact Model Intercomparison Project (ISI--MIP): project framework. *Proceedings of the National Academy of Sciences* 2014; **111**(9): 3228-32.
8. Taylor KE, Stouffer RJ, Meehl GA. An overview of CMIP5 and the experiment design. *Bulletin of the American Meteorological Society* 2012; **93**(4): 485-98.
9. IPCC. Climate Change 2014: Synthesis Report. Contribution of Working Groups I, II and III to the Fifth Assessment Report of the Intergovernmental Panel on Climate Change. Geneva: IPCC, 2014.
10. Dunne JP, John JG, Adcroft AJ, et al. GFDL's ESM2 Global Coupled Climate-Carbon Earth System Models. Part I: Physical formulation and baseline simulation characteristics. *Journal of Climate* 2012; **25**(19): 6646-65.
11. Dunne JP, John JG, Shevliakova E, et al. GFDL's ESM2 Global Coupled Climate-Carbon Earth System Models. Part II: Carbon system formulation and baseline simulation characteristics. *Journal of Climate* 2013; **26**(7): 2247-67.
12. Jones CD, Hughes JK, Bellouin N, et al. The HadGEM2-ES implementation of CMIP5 centennial simulations. *Geoscientific Model Development* 2011; **4**(3): 543-70.
13. Mignot J, Bony S. Presentation and analysis of the IPSL and CNRM climate models used in CMIP5. *Climate Dynamics* 2013; **40**: 2089.
14. Watanabe S, Hajima T, Sudo K, et al. MIROC-ESM 2010: model description and basic results of CMIP 5-20c3m experiments. *Geoscientific Model Development* 2011; **4**(4): 845-72.
15. Bentsen M, Bethke I, Debernard JB, et al. The Norwegian Earth System Model, NorESM1-M - Part 1: Description and basic evaluation. *Geoscientific Model Development Discussions* 2012; **5**: 2843-931.
16. Iversen T, Bentsen M, Bethke I, et al. The Norwegian Earth System Model, NorESM1-M - Part 2: climate response and scenario projections. *Geoscientific Model Development Discussions* 2013; **6**(2): 389-415.
17. Hempel S, Frieler K, Warszawski L, Schewe J, Piontek F. A trend-preserving bias correction - the ISI-MIP approach. *Earth System Dynamics* 2013; **4**(2): 219-36.
18. Raisanen J, Raty O. Projections of daily mean temperature variability in the future: cross-validation tests with ENSEMBLES regional climate simulations. *Climate dynamics* 2013; **41**(5-6): 1553-68.
19. Keellings D. Evaluation of downscaled CMIP5 model skill in simulating daily maximum temperature over the southeastern United States. *International Journal of Climatology* 2016: DOI: 10.1002/joc.4612.
20. Bhaskaran K, Gasparrini A, Hajat S, Smeeth L, Armstrong B. Time series regression studies in environmental epidemiology. *International Journal of Epidemiology* 2013; **42**(4): 1187-95.
21. Gasparrini A. Modeling exposure-lag-response associations with distributed lag non-linear models. *Statistics in Medicine* 2014; **33**(5): 881-99.
22. Gasparrini A, Armstrong B. Reducing and meta-analyzing estimates from distributed lag non-linear models. *BMC Medical Research Methodology* 2013; **13**(1): 1.
23. Gasparrini A, Armstrong B, Kenward MG. Multivariate meta-analysis for non-linear and other multi-parameter associations. *Statistics in Medicine* 2012; **31**(29): 3821-39.

24. Higgins JPT, Thompson SG. Quantifying heterogeneity in a meta-analysis. *Statistics in Medicine* 2002; **21**(11): 1539-58.
25. Gasparrini A, Leone M. Attributable risk from distributed lag models. *BMC Medical Research Methodology* 2014; **14**(1): 55.
26. Arbuthnott K, Hajat S, Heaviside C, Vardoulakis S. Changes in population susceptibility to heat and cold over time: assessing adaptation to climate change. *Environmental Health* 2016; **15**(Suppl 1): 33.
27. Nordio F, Zanobetti A, Colicino E, Kloog I, Schwartz J. Changing patterns of the temperature–mortality association by time and location in the US, and implications for climate change. *Environment International* 2015; **81**: 80-6.
